# L-Amino Acids Elicit Diverse Response Patterns in Taste Sensory Cells: A Role for Multiple Receptors

**DOI:** 10.1371/journal.pone.0130088

**Published:** 2015-06-25

**Authors:** Shreoshi Pal Choudhuri, Rona J. Delay, Eugene R. Delay

**Affiliations:** Department of Biology and Vermont Chemosensory Group, The University of Vermont, Burlington, Vermont, United States of America; Barnard College, Columbia University, UNITED STATES

## Abstract

Umami, the fifth basic taste, is elicited by the L-amino acid, glutamate. A unique characteristic of umami taste is the response potentiation by 5’ ribonucleotide monophosphates, which are also capable of eliciting an umami taste. Initial reports using human embryonic kidney (HEK) cells suggested that there is one broadly tuned receptor heterodimer, T1r1+T1r3, which detects L-glutamate and all other L-amino acids. However, there is growing evidence that multiple receptors detect glutamate in the oral cavity. While much is understood about glutamate transduction, the mechanisms for detecting the tastes of other L-amino acids are less well understood. We used calcium imaging of isolated taste sensory cells and taste cell clusters from the circumvallate and foliate papillae of C57BL/6J and T1r3 knockout mice to determine if other receptors might also be involved in detection of L-amino acids. Ratiometric imaging with Fura-2 was used to study calcium responses to monopotassium L-glutamate, L-serine, L-arginine, and L-glutamine, with and without inosine 5’ monophosphate (IMP). The results of these experiments showed that the response patterns elicited by L-amino acids varied significantly across taste sensory cells. L-amino acids other than glutamate also elicited synergistic responses in a subset of taste sensory cells. Along with its role in synergism, IMP alone elicited a response in a large number of taste sensory cells. Our data indicate that synergistic and non-synergistic responses to L-amino acids and IMP are mediated by multiple receptors or possibly a receptor complex.

## Introduction

The sense of taste provides vital sensory information to determine whether a particular food or beverage will be ingested. It is integral for regulating normal ingestive decisions and is particularly important to people experiencing any disease conditions such as obesity, diabetes, hypertension, coronary artery disease, anorexia, and malnutrition [1–11]. Detection of taste stimuli is mediated by the coordinated actions of distinct types of taste sensory cells (TSCs) housed in taste buds of specialized papillae in the oral cavity. Taste receptors in TSCs that detect compounds eliciting sweet, salty, sour, bitter, and umami tastes are the key players in selecting nutrients. One such example is amino acids that are an important part of one’s diet.

Each basic taste quality generally signals a fundamental type of nutrient. For example, sweet taste is often considered a general signal for carbohydrates in food whereas umami taste is thought to signal the presence of proteins and nucleotides. Umami taste is characterized by two distinctive qualities: 1) a unique savory taste, and 2) synergism with 5’ nucleotide monophosphates, especially inosine 5’ monophosphate (IMP) and guanosine 5’ monophosphate (GMP) [12, 13]. The prototypical compound that elicits umami taste in humans is monosodium glutamate (MSG), a substance known to increase the palatability of food [14–16]. Recent research has shown that fortification of meals with an appropriate amount of MSG may improve food intake and therefore has potential for improving nutritional status and quality of life in elderly and nutritionally deficient patients [16–19]. Thus, understanding the receptors and transduction pathways that mediate umami taste could be beneficial in regulating the intake of nutrients that are critical for clinical populations with dietary challenges.

Umami compounds are detected by receptors expressed in Type II TSCs [20–25]. A long standing question concerning umami taste relates to whether umami and L-amino acids are detected by one receptor or multiple receptors. Previous studies including *in vitro* receptor expression, behavioral, nerve recording, and single cell recording experiments have suggested that members of the T1r receptor family form a heterodimer, T1r1+T1r3, which is an umami receptor in mice [23, 24]. Further support for its role as an umami receptor comes from studies with knockout (KO) mice in which *Tas1r1* or *Tas1r3* gene was selectively eliminated. Some of these studies have shown that these mice lose all ability to respond to umami stimuli [25]. However, other studies with independently derived T1r1 and T1r3 receptor KO mice found only partial taste loss for umami [22, 26, 27]. Additional studies have reported that other G-protein coupled receptors (GPCRs) such as truncated variants of mGluR4 (taste-mGluR4) and mGluR1 (taste-mGluR1), as well as the brain versions of mGluR4 and 1 may be involved in the detection of umami compounds [20, 21, 28–30]. Moreover, there is evidence for expression of mGluR2 and mGluR3 in taste buds [31]. Together these studies argue for the involvement of more than one receptor that can detect umami compounds.

While much is known about glutamate transduction, detection mechanisms of other L-amino acids are less well understood. Understanding the receptor system and transduction mechanisms for L-amino acids is noteworthy because L-amino acids function as the building blocks of proteins and as metabolic fuel. Having more than one receptor for detecting these compounds would be advantageous. One such candidate receptor is the T1r1+T1r3 heterodimer. Transfected human embryonic kidney (HEK) cell expression data suggest that the murine heterodimer T1r1+T1r3 is a broadly tuned L-amino acid receptor [24]. Behavioral data suggest that one or more mGluR receptors may also detect some amino acids [32, 33]. Although IMP potentiates the response for several L-amino acids in HEK cells, some L-amino acids could elicit a response only in the presence of IMP [24]. Like umami, this property makes understanding L-amino acid detection mechanisms particularly important as they could be targets for altering taste properties of food, making it more or less desirable.

If a single receptor is involved in the detection of all L-amino acids, then all L-amino acids should have the same or very similar taste properties. However, not all L amino acids elicit the same taste. Some L-amino acids are attractive to rodents, some are aversive. Human psychophysical studies have showed that at low concentrations, L-serine (Ser) and L-glutamine (Gln) elicit a sensation that is mainly sweet, whereas L- arginine (Arg) is bitter. Further, at high concentrations, Ser and Gln elicit an umami taste [34, 35]. Additional studies with rats have shown that rats are differentially sensitive to MSG, Ser, and Arg [33]. Collectively, these data suggest the possibility of multiple L-amino acid receptors.

In this study, we investigated TSCs from mice circumvallate and foliate papillae located at the posterior portion of the tongue, to explore the nature of the responses and potential receptors involved in detection of L-amino acids. We focused on the posterior portion of the tongue for several reasons. First, the posterior portion of the tongue has been shown to generate a strong response to umami and L-amino acid stimuli [36]. Second, the circumvallate and foliate papillae are much richer in TSCs compared to the fungiform papillae. Third, the pattern of expression of different receptors varies between the posterior and anterior portion of the tongue. Since this might contribute to differences in response patterns, we chose to study TSCs in the posterior part of the tongue to reduce potential sources of variability and enhance our ability to identify response patterns across TSCs. We used calcium (Ca^2+^) imaging of isolated TSCs and taste cell clusters to determine if: 1) single TSCs are responsive to a set of four L-amino acids, with and without IMP, and to IMP alone, 2) TSCs respond synergistically to the MIX of L-amino acid+IMP, and 3) TSCs of T1r3 KO mice can detect L-amino acids and respond synergistically in presence of IMP. We found that the response patterns elicited by L-amino acids varied significantly across TSCs. L-amino acids other than glutamate also elicited synergistic responses in a subset of TSCs. Along with its role in synergism, IMP itself also elicited a response in TSCs. Our study suggests that in addition to the T1r1+T1r3 heterodimer, another receptor or possibly receptor complex is/are involved in the detection of L-amino acids and IMP.

## Materials and Methods

### Ethical consideration

All experimental procedures were reviewed and approved by the University of Vermont’s Institutional Animal Care and Use Committee (IACUC protocol: 10–038). Mice were euthanized by CO_2_ asphyxiation followed by cervical dislocation. All efforts were made to minimize suffering.

### Animals

Male and female (>8 weeks old) C57BL/6J (WT) (Jackson labs), T1r3-GFP [37], and T1r3 KO [22] mice were used in this study. T1r3-GFP mice express enhanced green fluorescent protein (eGFP) under control of the *Tas1r3* gene promoter and were generated on C57BL/6J background. The T1r3-GFP mice were primarily used in the early phases of the study to help identify isolated TSCs. T1r3KO mice were generated on C57BL/6J background, and all 6 exons for the *Tas1r3* gene were eliminated [22]. Breeding stock for T1r3-GFP and T1r3 KO mice were generously donated by Dr. Robert Margolskee [22, 37]. GFP expression and genetic deletion of *Tas1r3* gene were verified by polymerase chain reaction (PCR). For clarity, throughout the paper we are using T1r1 and T1r3 to refer to the receptor proteins in mice. All mice were maintained on a 12-h light/12-h dark cycle with food and water provided *ad libitum*.

### Solutions

Tyrode’s solution contained (in mM): NaCl 140, KCl 5, MgCl_2_ 1, CaCl_2_ 2, HEPES 10, Glucose 10, and Na pyruvate 1. High potassium (high K^+^) Tyrode's solution contained the same constituents as regular Tyrode’s solution with the exception that 65 mM KCl was substituted for equimolar NaCl. Ca^2+^/ Mg^2+^ free Tyrode’s contained (in mM): NaCl 140, KCl 5, HEPES 10, Glucose 10, Na pyruvate 1, and EGTA 2. L-amino acids used as test solutions were (in mM): L-Arg 10, L-Ser 20, L-Gln 10, and monopotassium L-glutamate (MPG) 10. MPG was used to ensure that responses were not due to the sodium component of MSG. In many studies MPG has been successfully used to reliably evoke taste responses to the glutamate moiety [38, 39]. Additionally, 10mM of K^+^ is not sufficient to cause the amount of depolarize required to activate L-type Ca^2+^ channels expressed in TSCs. Di-sodium inosine 5’ monophosphate (IMP) was used at 1mM. The addition of 2mM sodium associated with IMP was very small compared to the amount of Na^+^ (140mM) in the bath and thus unlikely to elicit any cellular responses. Physiologically relevant stimulus concentrations for each substance were chosen from behavioral and physiological data to ensure that each concentration was above recognition threshold in rodents (mice and rats) [28, 36, 40, 41], but not high enough to cause any osmotic changes. The artificial sweetener, SC45647 (2-[[[[4-(aminomethyl)phenyl]amino]-[[(1*R*)-1-phenylethyl]amino]methyl]amino]ethane-1,1-diol) (100μM) was used as a sweet stimulus [42], and denatonium (2mM) or a mixture of cycloheximide (20μM) and denatonium (2mM) was used as a bitter stimulus. The L-amino acids, sweet, and bitter compounds were dissolved in Tyrode’s solution and made fresh every day. When generating a mixture solution (MIX) of L-amino acid+IMP, the concentration of each compound was the same as those used for the individual compounds. In the MIX, 1mM IMP was mixed with 10mM of either of MPG, Arg, Gln, or 20mM of Ser. In the AA-MIX (L-amino acid-MIX), all four L-amino acids (MPG, Ser, Arg, and Gln) were used at the same concentration as mentioned before. All solutions were adjusted to approximately pH 7.4 using NaOH or HCl.

### Taste cell isolation

Taste cells from circumvallate and foliate taste buds were isolated using a protocol adapted from Behe et al. [43] and Gilbertson et al. [44]. In short, mice were euthanized by CO_2_ asphyxiation followed by cervical dislocation. Tongues were removed and immersed in ice cold Tyrode’s solution. The lingual epithelium was removed by injecting an enzyme cocktail containing 0.8 mg/mL collagenase A (Sigma, St. Louis, MO), 1.5 mg/mL dispase II (Roche, Indianapolis, IN), 1 mg/mL trypsin inhibitor (Sigma, St. Louis, MO), and 0.05mg/mL elastase (Worthington, Lakewood, NJ) directly under the epithelium. The tongue was then incubated in Tyrode’s solution for 20 min followed by incubation in Ca^2+^/ Mg^2+^-free Tyrode’s for another 20 min. In both solutions, oxygen was supplied continuously. The epithelium was gently removed from the underlying connective tissue and pinned flat with the epithelium surface down on a sylgard-lined petri dish. The tissue was incubated in the enzyme cocktail (without dispase II) for 5 min before being transferred to Ca^2+^/Mg^2+^ free Tyrode’s solution for 20–25 min. Taste buds and TSCs were removed from circumvallate and foliate papillae by gentle suction using fire polished glass micropipettes. TSCs were plated into a shallow recording chamber with a glass cover-slip pre-coated with Concanavalin A (Sigma, St. Louis, MO) to promote cell adherence. This protocol enabled us to reliably obtain both isolated taste cells and clusters of taste cells. Typically the cells were viable for 6–7 hours.

### Calcium (Ca^2+^) imaging

Measurements of intracellular Ca^2+^ were obtained using the ratiometric fluorescent dye fura-2 AM (Molecular probes, Invitrogen Corporation, NY). Taste cells were incubated in 5μM fura-2, AM and 0.05% pluronic F-127 dissolved in DMSO in Tyrode’s solution for 25–30min. The recording session began after bath washing the cells in Tyrode’s solution for 10–20min. Images were acquired using an inverted fluorescent Nikon TE2000S microscope and C4742-95 digital camera. All solutions were bath applied using a gravity flow perfusion system. Stimuli were applied for 30s before returning to Tyrode's solution. Application of stimuli was in random order to avoid any systemic error. Sometimes a MIX of L-amino acid+IMP was applied after the L-amino acid, and sometimes the MIX was applied before the L-amino acid. In both instances we found some cells that elicited synergistic responses. Thus synergistic or non-synergistic responses were not dependent on the stimulus application sequence. At the end of any stimulus application, cells were washed in Tyrode’s solution for 5 to 9 min. We performed a desensitization study in which single cells were stimulated with the same stimulus 4 to 5 times with varying wash times in between. We found that a wash of 5 to 9 min between stimulus applications was optimal for repeated responses of similar magnitude, although in some cells desensitization occurred independent of an extended wash. If the final stimulus application in the test sequence did not elicit a response, we stimulated the cell with a compound which previously elicited a response to make sure that the cell was still alive. Images were captured every 3 s during stimulus application and every 5 to 15 s during wash. In order to minimize cell damage during long wash periods, we limited image capturing during washes. After a response, images were captured only until the Ca^2+^ level went back to baseline (~2–3 min after the start of stimulus application) and image capturing was resumed at least 1 min prior to next stimulus application. Fura2 AM was doubly excited at 340nm and 380nm and its emissions were recorded at 510nm. Simple PCI 6.0 software (Hamamatsu, Sewickley, PA) running on a PC computer was used to capture images. Changes in Ca^2+^ concentrations are reported as F340/F380 plotted over time after background subtraction.

### Quantification of calcium responses

Increases in intracellular Ca^2+^evoked by stimulus application were calculated as follows:

$$\Delta F=\frac{F_{Peak}-F_{Baseline}}{F_{Baseline}}*100\%$$

where ΔF is the percent change above baseline, F_Peak_ is the largest F340/380 ratio within 1 min following the onset of a stimulus application, and F_Baseline_ is the average of the five sampled F340/380 ratios immediately before stimulus application. Our criteria for considering ΔF a response were: 1) the same stimulus elicited an increase in intracellular Ca^2+^ at least twice, and 2) each ΔF≥5%. Furthermore, ΔF was considered a synergistic response when:

$$\Delta F_{MIX}>\Delta F_{AA}+\Delta F_{IMP}$$

where ΔF_MIX_ is the percent change above baseline following application of a MIX of L-amino acid (where L-amino acid can be MPG, Ser, Arg, or Gln)+IMP, ΔF_AA_ is the percent change above baseline following application of MPG, Ser, Arg, or Gln alone, and ΔF_IMP_ is the percent change above baseline following application of IMP alone. Although peak Ca^2+^ is typically used to identify synergistic responses, we recognized that synergy might appear as an increase in duration of the signal instead of an increase in peak amplitude of the signal. To determine if we missed a large subset of potential synergistic responses, we measured the area under the curve of the responses classified as non-synergistic by peak amplitude. Only 2 cells from the Arg set and 3 cells from the Gln set were identified as potentially synergistic using the integrated response. Since these responses might also represent continued stimulation by residual stimulus solution rather than synergy, they were excluded from the analyses involving synergy in favor of the more reliable measure of peak amplitude.

## Results

### Single TSCs from WT mice respond to multiple, but not all L-amino acids

To better understand how TSCs respond to L-amino acids, and to determine if the T1r1+T1r3 heterodimer is the only receptor involved in L-amino acid detection, we investigated whether a single TSC would show a Ca^2+^ response to an array of L-amino acids with and without IMP. We isolated single TSCs and taste cell clusters from mouse circumvallate and foliate papilla. Each TSC was stimulated with 9 different stimuli: (1) MPG, (2) Ser, (3) Arg, (4) Gln, (5) IMP, (6) MPG+IMP, (7) Ser+IMP, (8) Arg+IMP, and (9) Gln+IMP. All test solutions were applied regardless of whether the cell responded to any individual stimulus. A total of 600 out of 1217 (49%) TSCs were successfully tested with all 9 stimuli, where the cells were alive until the end of the experiment. At least one stimulus was capable of eliciting a Ca^2+^ response (i.e. ΔF≥5% in response to stimulation) in 170 of 600 (28%) TSCs. Thus we focused our analysis on the 170 cells that had a Ca^2+^ response to at least one stimulus.

Like previous studies [25, 36, 45], we found that TSCs responded to L-amino acids when presented with IMP. Out of 170 responsive TSCs, 133 (78%) responded to MPG+IMP, 118 (69%) responded to Ser+IMP, 111(65%) responded to Arg+IMP, and 114 (67%) responded to Gln+IMP. TSCs also responded to the L-amino acids MPG, Ser, Arg, and Gln when presented individually (Table 1).

**Table 1:** Summary of responsive WT and T1r3 KO TSCs to the 9 stimuli.

	WT		T1r3 KO	
	Total number of cells successfully tested with all 9 stimuli: 600		Total number of cells successfully tested with all 9 stimuli: 154	
	Number of cells with response to any stimulus: 170 (28%)		Number of cells with response to any stimulus: 24 (16%)	
Stimulus	No. of Responsive cells out of 170 cells (%)	No. of Synergistic Cells	No. of Responsive cells out of 24 cells	No. of Synergistic Cells
IMP	121 (71)	N/A	14 (58)	N/A
MPG	78 (46)	N/A	16 (67)	N/A
MPG+IMP	133 (78)	87	21 (87)	9
Ser	65 (38)	N/A	24 (100)	N/A
Ser+IMP	118 (69)	59	24 (100)	15
Arg	63 (37)	N/A	18 (75)	N/A
Arg+IMP	111 (65)	70	23 (96)	14
Gln	66 (39)	N/A	18 (75)	N/A
Gln+IMP	114 (67)	75	16 (67)	2

Values are number of cells. Values in parenthesis are percentages.

**Fig 1:**
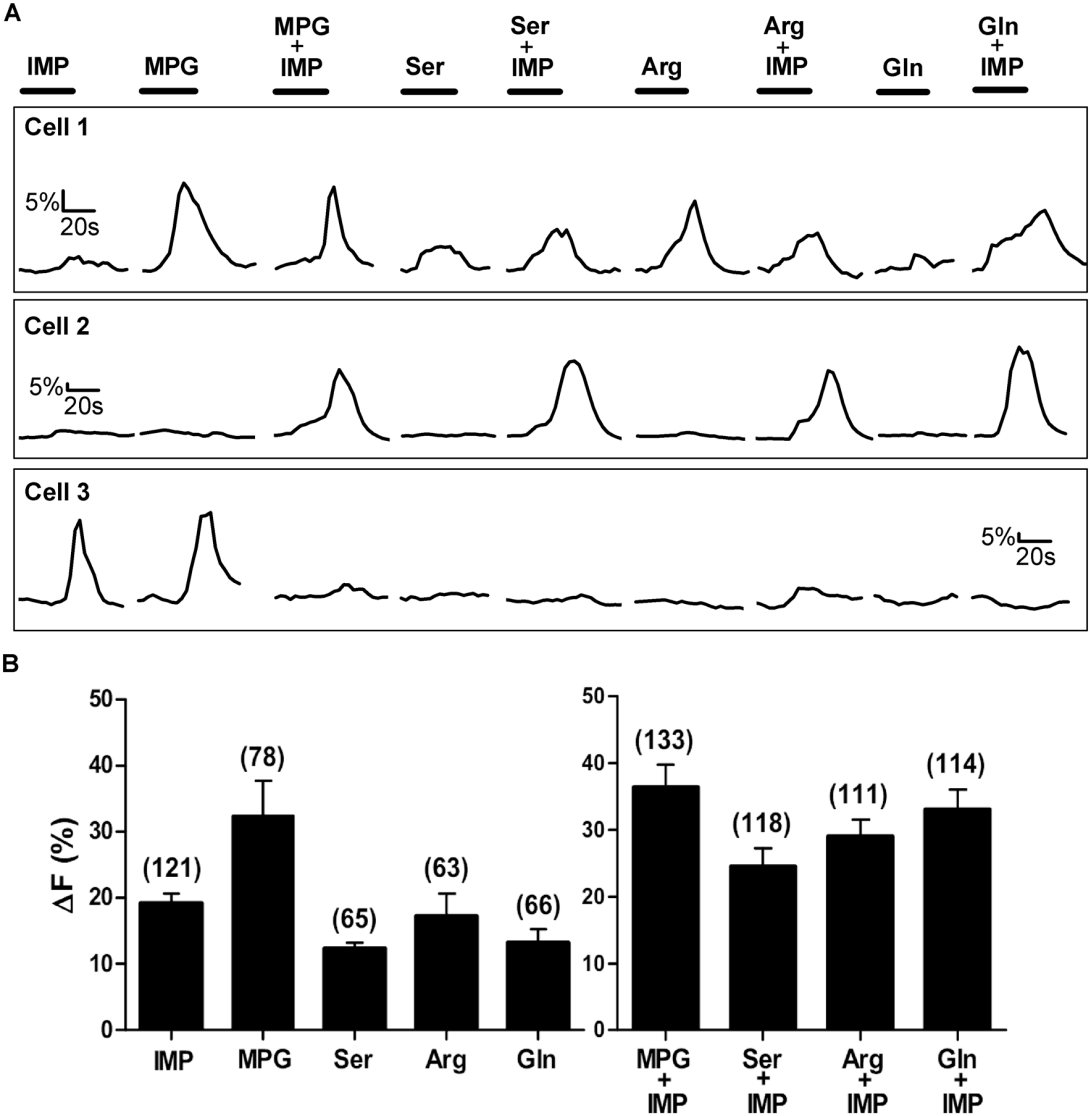
Representative Ca^2+^ responses of TSCs from WT mice. Stimuli tested were IMP (1mM), 4 L-amino acids from different side-chain groups (MPG (10mM), Ser (20mM), Arg (10mM), and Gln (10mM)), and L-amino acids with IMP. [A] Ca^2+^ responses of 3 sample TSCs. Each cell was tested with all 9 stimuli. The responses of the three cells are examples of some of the different response patterns to the array of stimuli. The bar above each stimulus trace represents stimulus application time (30 sec). [B] Mean±SEM amplitude of Ca^2+^ increase above baseline for responsive cells only, i.e. cells with a change in Ca^2+^ ≥5%. Numbers in parenthesis are the number of cells.

In previous research, HEK cells transiently transfected with the T1r1+T1r3 heterodimer, exhibited Ca^2+^ responses to the umami compound L-glutamate, but only when IMP was present. Notably, IMP alone had no effect on HEK cells [24]. In contrast, we found that MPG presented alone elicited Ca^2+^ responses in 78 of 170 (46%) responsive TSCs. The mean amplitude of MPG (10mM) evoked Ca^2+^ response (ΔF) was 32.38±5.3% (Mean±SEM) above baseline (Table 1; Fig 1A Cell 1 and Cell 3, Fig 1B). In addition, IMP presented alone elicited a Ca^2+^ response of 19.20±1.48% (Mean±SEM) above baseline in 121 of 170 (71.17%) responsive TSCs (Table 1; Fig 1A Cell 3, Fig 1B).

In our study, a single TSC when stimulated with 9 different stimuli, often responded to more than one L-amino acid but did not necessarily respond to all of the test solutions. For example, cell 1 in Fig 1A, responded to all 9 stimuli tested, whereas cell 2 in Fig 1A responded to IMP and to the L-amino acids only when presented with IMP. Clearly, cell 2’s responses to each of the L-amino acid+IMP-MIXes were not elicited solely by IMP, as response magnitudes to the MIXes (35–45% above baseline) were much greater than the response magnitude to IMP (5% above baseline) alone.

Only 10 of 170 (6%) TSCs, responded to all 9 stimuli, while the remaining TSCs responded to some but not all stimuli. Moreover, TSCs did not always respond to L-amino acids when presented with IMP (Table 1; Fig 1). Analyzing TSCs with responses to only L-amino acids, irrespective of their responses to IMP or any MIX of L-amino acid with IMP, we found 21 of 170 (12%) TSCs responded only to MPG but not to any of the other three L-amino acids tested (Ser, Arg, and Gln). Another 57 of 170 (33%) TSCs responded to MPG and to one or more of the other three L-amino acids tested. On the other hand, 54 of 170 (31%) TSCs did not respond to MPG but did respond to one or more of the other three L-amino acids. These results suggest that all L-amino acid responsive cells do not necessarily respond to the prototypical umami L-amino acid, glutamate. In addition, more than three-fourths of the TSCs responded to 1mM IMP, suggesting that IMP may be detected by a mechanism that is independent of the T1r1+T1r3 heterodimer.

### In some but not all cases, L-amino acids elicit synergy when presented with IMP

Synergy between 5’ ribonucleotides (IMP and GMP) and L-glutamate is a defining characteristic of umami taste. The basis for this effect begins in the TSC and the taste bud. Previous studies [25, 36, 45] have shown that MPG elicited synergistic responses in TSCs when mixed with IMP or GMP. In addition to increasing response intensity, GMP also increased the number of responsive TSCs [45]. Similar to previous studies, we found a greater number of TSCs responsive to the MIX of MPG+IMP. For example, 70% more TSCs responded to MPG in presence of IMP than to MPG alone. In the same way, IMP also increased the number of cells responding to other L-amino acids. For instance, 81%, 76%, and 72% more cells responded to Ser, Arg, and Gln, respectively, in the presence of IMP compared to the L-amino acid alone (Table 1). We next analyzed the peak amplitude of MIX (L-amino acid+IMP) responsive TSCs to determine which Ca^2+^ responses were synergistic and if L-amino acids other than glutamate also elicited synergistic responses. Of the 170 TSCs, 133 (78%) TSCs responded to the MPG+IMP-MIX, 118 (69%) TSCs responded to the Ser+IMP-MIX, 111(65%) TSCs responded to the Arg+IMP-MIX, and 114 (67%) TSCs responded to the Gln+IMP-MIX.

**Fig 2:**
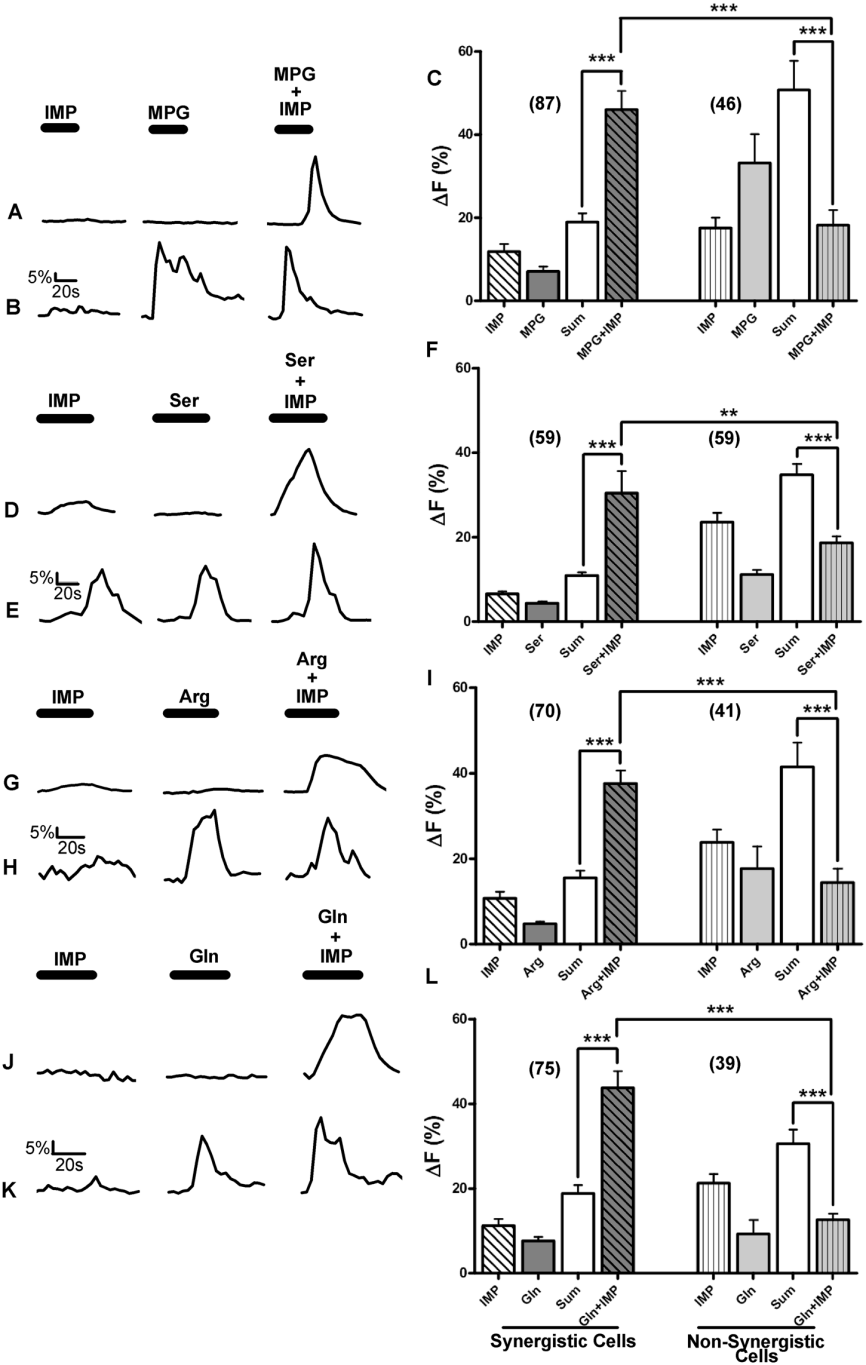
Some but not all TSCs generated synergistic response to the L-amino acid+IMP MIXes. Ca^2+^ responses of WT TSCs to the four L-amino acid sets are shown. Each L-amino acid set consisted of one of the four different L-amino acids (MPG (10mM), Ser (20mM), Arg (10mM), or Gln (10mM), respectively), IMP (1mM), and the MIX of L-amino acid+IMP. [A, D, G, J] Representative TSC responses where the magnitudes of MIX responses were greater than the summation of individual L-amino acid and IMP responses, i.e., the MIX responses were synergistic. [B, E, H, I] Representative TSC responses where the magnitudes of MIX responses were greater than or equal to the responses of the L-amino acid or IMP individually, but not greater than the summation of individual L-amino acid and IMP responses, i.e., the MIX responses were not synergistic. [C, F, I, L] A Mean±SEM response for the L-amino acid sets exhibiting synergistic and non-synergistic responses. For each L-amino acid set, the MIX responses generated by synergistic cells were significantly greater than the calculated sum (Sum) of L-amino acid and IMP responses. For non-synergistic cells, MIX responses were significantly smaller than the calculated sums of responses to L-amino acid and IMP. MIX responses of synergistic cells were also significantly greater than the MIX responses of non-synergistic cells. Numbers in parenthesis are the number of cells. One-way ANOVA followed by Bonferroni post hoc tests were used for statistical comparisons. ***P<0.0001, **P<0.001.

For each of the L-amino acids tested, only a subset of MIX-responsive cells elicited synergistic responses (Table 1; Fig 2). Approximately 50–65% of the MIX-responsive TSCs responded synergistically to one or more of the amino acids. Of the 133 MPG+IMP-MIX-responsive TSCs, 87 (65%) cells showed synergistic responses. Clearly, not all MPG+IMP-MIX-responsive cells were synergistic (Table 1). For synergistic responses, the average increase in intracellular Ca^2+^ response to MIX was significantly greater than the sum of the responses to the individual stimulus compounds (One way ANOVA; P<0.0001). In addition, the mean Ca^2+^ increase for synergistic responses of these cells was significantly greater than the MIX response of non-synergistic cells (One way ANOVA; P<0.0001) (Fig 2). We similarly analyzed the Ca^2+^ responses to the MIXes of Ser+IMP, Arg+IMP, and Gln+IMP. Of the 118 Ser+IMP-MIX-responsive cells, 59 (50%) cells exhibited synergistic responses. Likewise, 70 of 111 (63%) Arg+IMP-MIX-responsive cells, and 75 of 114 (65%) Gln+IMP-MIX-responsive cells showed synergistic responses (Table 1). Like the MPG+IMP-MIX-responsive TSCs, the magnitudes of the Ca^2+^ responses of TSCs that responded synergistically to the MIX for each of the L-amino acids were significantly greater than MIX responses of non-synergistic cells (Fig 2).

### MIX-Responsive TSCs respond differently to IMP and L-Amino Acids

To further differentiate L-amino acid response patterns, we analyzed TSC responses to individual L-amino acid set, i.e., IMP and L-amino acid with and without IMP. Each L-amino acid set consisted of one of the four L-amino acids (MPG, Ser, Arg, and Gln), IMP, and the MIX (the L-amino acid+IMP). Each set of MIX-responsive TSCs was subdivided according to their responsiveness to the individual components of the MIX. For the MPG set (Table 2), which consisted of MPG, IMP, and MPG+IMP stimuli, 133 of 170 (78%) TSCs responded to the MPG+IMP-MIX. Of these 133 cells, a subset of 23 (17%) TSCs responded only to the MIX, but not to MPG or IMP presented individually, and 100% (23 out of 23 cells) of those responses were synergistic. On the other hand, another subset of 20 out of the 133 (15%) MIX-responsive cells responded to MPG but not IMP, and 8 of those 20 (40%) cells exhibited synergistic responses. Furthermore, another subset of 45 of the 133 (34%) MPG+IMP-responsive cells responded to IMP but not to MPG alone. Of these 45 TSCs, 30 (66%) TSCs responded synergistically. Lastly, a different subset of 45 TSCs of 133 MPG+IMP-responsive cells also responded to both IMP and MPG when presented alone. Of these 45 cells, 26 (58%) cells demonstrated a synergistic response to the MIX (Table 2). Similar response patterns were also found for the other three L-amino acid (Ser, Arg, and Gln) sets (Table 2). In summary, some TSCs responded to a MIX of an L-amino acid+IMP, but only a subset of those MIX-responsive cells was synergistic. Moreover, MIX-responsive TSCs may or may not respond to the individual components of the MIX. Additionally, clustering of MIX-responsive TSCs by their response to individual stimuli showed that almost all of the TSCs that did not respond to individual stimuli (i.e., L-amino acid or IMP), generated a synergistic response to the MIX.

**Table 2:** Summary of WT MIX-responsive synergistic and non-synergistic TSCs.

	**MPG+IMP-MIX-Responsive TSCs**		
Calcium Response	**Synergistic**	**Non-Synergistic**	
YES = **+**; No = **-**	Number of TSCs (%)		**Total**
MPG - / IMP -	23	0	**23 (17)**
MPG - / IMP +	30	15	**45 (34)**
MPG + / IMP -	8	12	**20 (15)**
MPG + / IMP +	26	19	**45 (34)**
**Total**	**87 (65)**	**46 (35)**	**133**
	**Ser+IMP-MIX-Responsive TSCs**		
Calcium Response	**Synergistic**	**Non-Synergistic**	
YES = **+**; No = **-**	Number of TSCs (%)		**Total**
Ser - / IMP -	13	0	**13 (11)**
Ser - / IMP +	32	14	**46 (39)**
Ser + / IMP -	7	7	**14 (12)**
Ser + / IMP +	7	38	**45 (38)**
**Total**	**59 (50.0)**	**59 (50.0)**	**118**
	**Arg+IMP-MIX-Responsive TSCs**		
Calcium Response	**Synergistic**	**Non-Synergistic**	
YES = **+**; No = **-**	Number of TSCs		**Total**
Arg - / IMP -	15	2	**17 (15)**
Arg - / IMP +	36	16	**52 (47)**
Arg + / IMP -	3	5	**8 (7)**
Arg + / IMP +	16	18	**34 (31)**
**Total**	**70 (63)**	**41 (37)**	**111**
	**Gln+IMP-MIX-Responsive TSCs**		
Calcium Response	**Synergistic**	**Non-Synergistic**	
YES = **+**; No = **-**	Number of TSCs		**Total**
Gln - / IMP -	14	2	**16 (14)**
Gln - / IMP +	23	23	**46 (40)**
Gln + / IMP -	6	1	**7 (6)**
Gln + / IMP +	32	13	**45 (40)**
**Total**	**75 (65)**	**39 (34)**	**114**

MIX-responsive TSCs responded differently to the individual components of each L-amino acid set. Values are number of cells. Values in parenthesis are percentages. +, response;-, no response to the stimulus. Note: For 2 cells in the Arg set, and 2 cells in the Gln set, MIX responses were not synergistic even though IMP nor the L-amino acid alone elicited a response (their increases in Ca^2+^ were <5% of baseline). For these cells, the increases in Ca^2+^ in response to IMP, and L-amino acids individually were between 2.5% and 3% above baseline, and thus were not considered to be responses. However, the MIX elicited very small responses that were just above 5% of baseline Ca^2+^, but not larger than the added sum of individual responses. Thus, these MIX responses were not synergistic. Since only around 2% of the cells showed this type of response, we did not include these cells in our discussion of synergy.

### Synergistic and non-synergistic responses are mediated by different receptors

**Fig 3:**
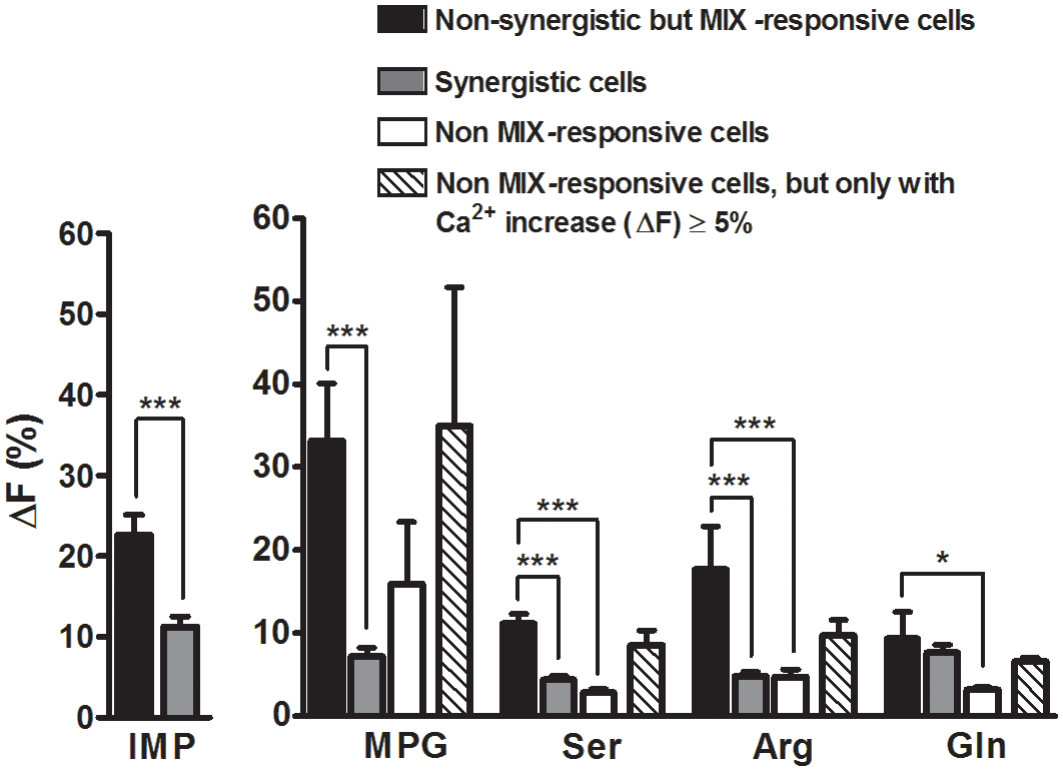
Ca^2+^ responses to L-amino acids and IMP in MIX-responsive and non-MIX-responsive cells. Bars represent Mean±SEM Ca^2+^ responses. Average Ca^2+^ responses to the individual stimulus components of the MIX were compared for two groups of MIX-responsive cells: 1) non-synergistic and 2) synergistic cells. The increases in intracellular Ca^2+^ in response to IMP and the L-amino acids were significantly smaller for synergistic cells (gray bars) compared to non-synergistic (black bars) MIX-responsive cells. Cells that did not respond to the MIX but responded to an L-amino acid (non-MIX-responsive cells; white bars) presented alone also generated Ca^2+^ responses with a similar magnitude as those of MIX-responsive non-synergistic cells (gray bars). To eliminate bias by cells that may not have a receptor, only those cells with Ca^2+^ responses (ΔF≥5%) to L-amino acids were analyzed (see results). For these cells, Ca^2+^ responses to individual L-amino acids were not significantly different from Ca^2+^ responses of MIX-responsive, non-synergistic cells (patterned bars). Unpaired t-test (for IMP), and One-way ANOVA followed by Bonferroni post hoc t-test (for L-amino acids) were used for statistical comparison. ***P<0.0001, **P<0.001, *P<0.05.

In recent years, evaluation of taste cell transduction mechanisms has focused mostly on whether or not TSCs exhibit any Ca^2+^ increase in response to stimulation, whereas much less attention has been given to the intensity of these responses. Since changes in response intensity related to synergy are likely to take place through mechanisms within the signal transduction pathway, we compared the average Ca^2+^ responses to the individual stimulus components of the MIX of two groups of MIX-responsive cells: 1) non-synergistic and 2) synergistic cells. Interestingly, increases in intracellular Ca^2+^ in response to IMP were significantly smaller for synergistic cells compared to non-synergistic cells (Unpaired t-test; P<0.0001) (Fig 3). Similarly, when stimulated with L-amino acids (MPG, Ser, or Arg), the intracellular Ca^2+^ responses to these L-amino acids were significantly smaller for synergistic cells compared to non-synergistic MIX-responsive cells (One way ANOVA; P<0.0001) (Fig 3). These response patterns suggest the involvement of more than one receptor. One possible scenario is that there are two types of receptors. One type of receptor may be involved in synergistic responses with no or small responses to individual components of the MIX. The second type of receptor may respond to the individual components of the MIX (L-amino acids or IMP), and any reaction to the MIX is solely a response to the components in the MIX.

Since synergistic TSCs had smaller responses to the individual components of a MIX than non-synergistic TSCs, we asked whether cells that do not respond to the MIX but respond to an L-amino acid (non-MIX-responsive cells) presented alone also generated Ca^2+^ responses with a similar magnitude as those of MIX-responsive non-synergistic cells. Surprisingly, for non-MIX-responsive cells, Ca^2+^ responses to individual L-amino acids were also significantly smaller than Ca^2+^ responses of MIX-responsive, non-synergistic cells (Fig 3). Cells that responded to IMP but not to the MIX or to the individual L-amino acid of the MIX may not express any receptor for L-amino acid binding, thereby skewing the whole population of data towards null responses. To avoid this problem, we considered only cells with Ca^2+^ responses (ΔF≥5%) to L-amino acids. As expected, these responses were not significantly different from non-synergistic MIX-responsive cells (Fig 3). This further supports the possibility that different receptors are probably involved in synergistic and non-synergistic responses.

### Some TSCs were broadly tuned

**Fig 4:**
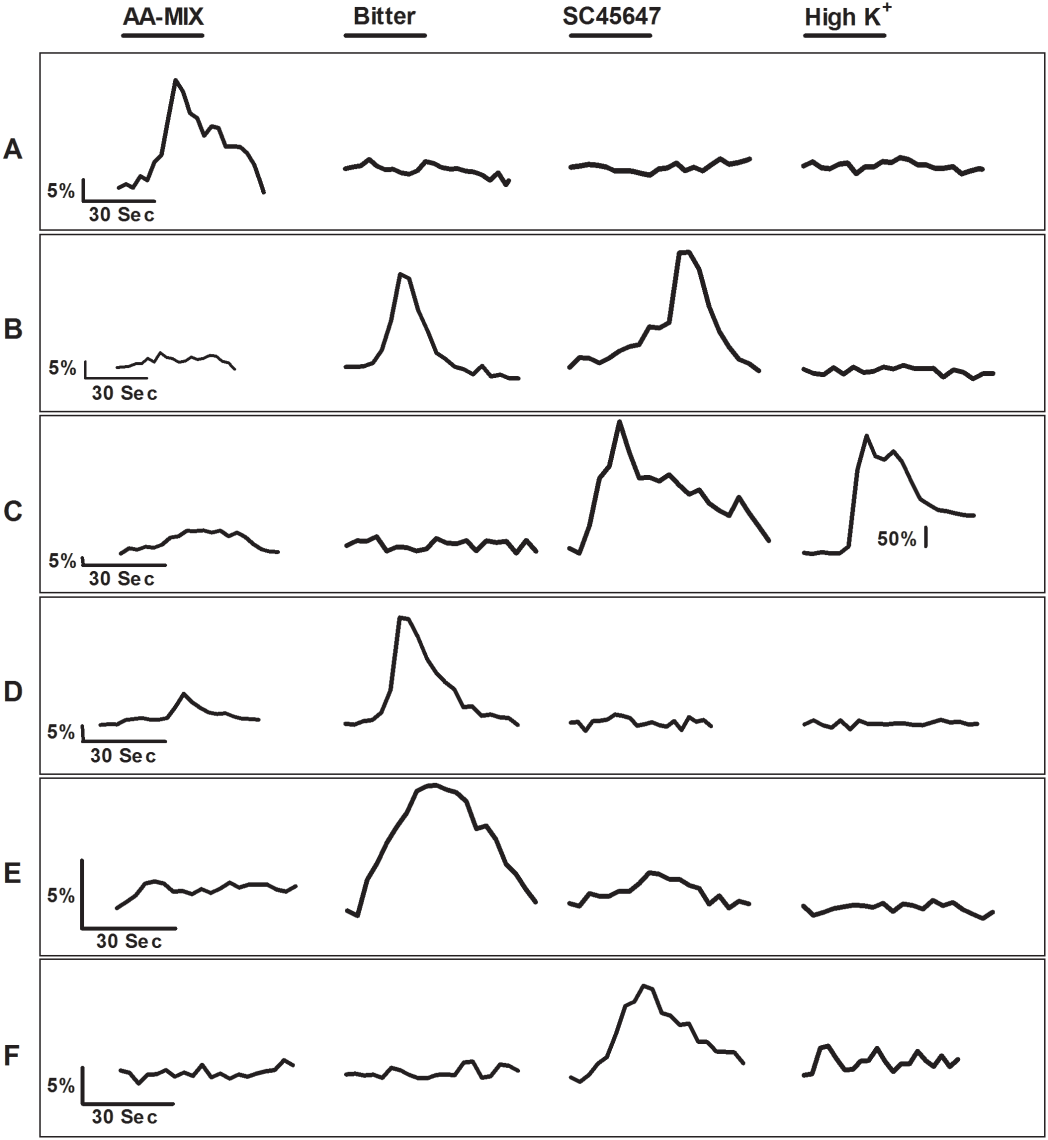
Representative Ca^2+^ responses elicited by TSCs during stimulation with 4 different stimuli. Cells were stimulated with 4 different stimuli, L-amino acid MIX (AA-MIX) (MPG (10mM), Ser (20mM), Arg (10mM), and Gln (10 mM)), sweet (SC45647 (100μM)), bitter (denatonium (2mM) or cycloheximide (20μM)+denatonium (2mM)), and high-K^+^ (65mM) solution. The bar above each stimulus trace represents the 30 s stimulus application period. [A] A TSC that responded only to AA-MIX. [B] A TSC that responded to bitter and sweet stimuli. [C] A TSC that responded to AA-MIX, sweet, and high-K^+^ solution. AA-MIX elicited a very small increase in cytosolic Ca^2+^ compared to sweet stimulus. The high-K^+^ solution elicited very large increase in cytosolic Ca^2+^, suggesting the presence to voltage-gated calcium channels. Note: A different Y axis scale was used for the high-K^+^ response as the high-K^+^ elicited response was much larger compared to AA-MIX or sweet responses. [D] This TSC responded to both AA-MIX and bitter stimuli. AA-MIX elicited a very small increase in cytosolic Ca^2+^ compared to bitter stimulus. [E] This TSC responded only to bitter stimuli. [F] This TSC responded only to sweet stimuli.

To further characterize the response-specificity of TSCs we tested additional TSCs to determine if they responded to L-amino acids as well as KCl, bitter and/or sweet, stimuli. In total, 135 isolated TSCs were tested with the mixture of L-amino acids (AA-MIX), sweet, and bitter stimuli. Of 135 cells, 13 (10%) cells responded to AA-MIX, 43 (31%) cells responded to bitter stimuli, and 26 (19%) cells responded to the sweet stimulus. The mean amplitude of Ca^2+^ increase (ΔF) for AA-MIX, bitter, and sweet stimuli were 40.26±15.13, 15.05±2.57, and 16.47±5.61 (Mean±SEM) above baseline, respectively. Of the 13 AA-MIX-responsive cells, 10 cells (77%) responded to the AA-MIX only (Fig 4A). The other 3 AA-MIX responsive TSCs also responded to sweet, bitter, or both of the stimuli (Fig 4B, 4C and 4D). Additionally, we tested 9 of these TSCs with the high-K^+^ solution to see if they were also responsive to high-K^+^. Of these 9 cells, only 1 isolated cell (11%) responded to high-K^+^ (Fig 4C). This cell was also responsive to sweet stimuli. Tomchik et al. [46] proposed that this cell type may be a pre-synaptic cell. On the other hand, Dando and Roper suggested that this type of response may be the result of cell-to-cell communication between Type II and Type III cell [47]. However, in our case the recording was obtained from an isolated cell, thus the response elicited by this cell cannot be influenced by another cell. It should also be noted that none of the cells that responded only to AA-MIX, responded to a high-K^+^ solution.

Although our focus was on the AA-MIX-responsive cells, we also evaluated these cells from the perspective of bitter and sweet responsiveness. Of the 43 bitter responsive cells, 24 cells (57%) responded to only bitter (Fig 4E), and of 26 sweet responsive cells, 7 cells (27%) responded to only sweet stimuli (Fig 4F). These data suggest that while some cells are narrowly tuned to a specific type of stimulus, at least a small proportion cells are broadly tuned to multiple types of taste stimuli. Our data are in agreement with previous studies, which also reported the presence of broadly tuned and narrowly tuned TSCs [46, 48, 49].

### TSCs from T1r3 KO mice responded to different L-amino acids

The taste receptor T1r1+T1r3 heterodimer functions as an umami and L-amino acid receptor in mice. To determine if T1r3 is an obligatory component for L-amino acid responses, TSCs from circumvallate and foliate papillae of T1r3 KO mice were tested with the same stimuli used to test TSCs from WT mice. We screened a total of 320 TSCs of T1r3 KO mice for responses and successfully tested 154 cells with all 9 stimuli. Ca^2+^ responses were detected from 24 of 154 (16%) cells. As a control, we compared the high-K^+^ induced responses of TSCs of WT and T1r3 KO mice. The incidence and magnitude of these responses were not significantly different between WT and T1r3 KO mice (WT, n = 20 out of 56 cells (36%); T1r3 KO, n = 23 out of 61 cells (38%); Unpaired t-test, P = 0.87).

**Fig 5:**
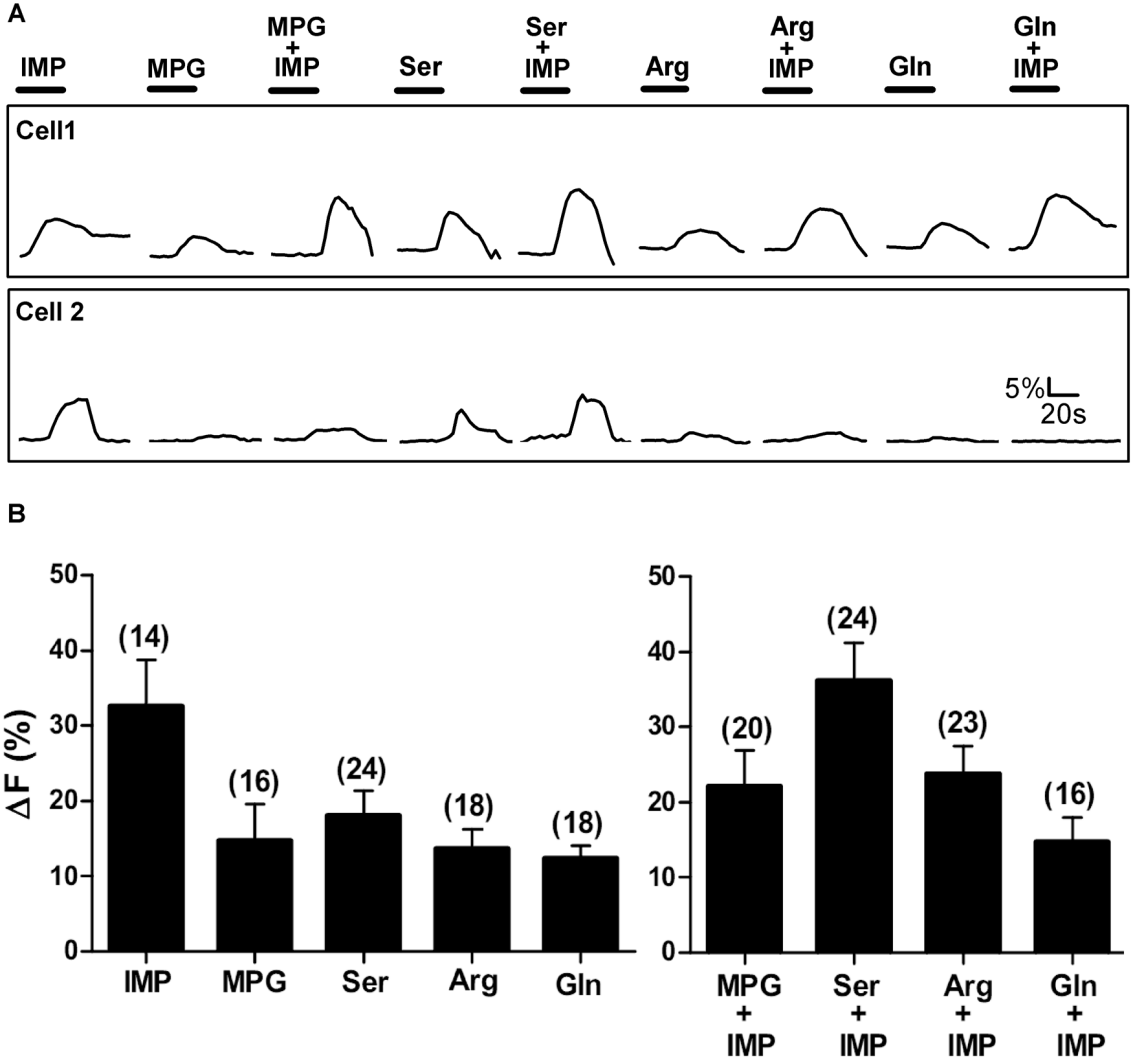
Representative Ca^2+^ responses of TSCs from T1r3 KO mice. Stimuli tested were IMP (1mM), 4 different L-amino acids (MPG (10mM), Ser (20mM), Arg (10mM), and Gln (10mM)), and L-amino acids with IMP. [A] Ca^2+^ responses of 2 sample TSCs. Each cell was tested with all 9 stimuli. The responses of these cells are examples of some of the different response patterns elicited by the array of stimuli. The bar above each stimulus trace represents the stimulus application time (30 sec). [B] Mean±SEM amplitude of Ca^2+^ increase (ΔF%) above baseline for responsive cells only, i.e. only cells with a change in baseline Ca^2+^≥5%. Numbers in parenthesis are the number of cells.

TSCs from T1r3 KO mice not only responded to L-glutamate but also responded to other L-amino acids (Table 1; Fig 5). Of the 24 responsive cells, MPG elicited an increase in Ca^2+^ of 14.6±4.9% (Mean±SEM) above baseline in 16 (67%) TSCs (Table 1; Fig 5). Ser, Arg, and Gln also elicited responses in 24 (100%), 18 (75%), and 18 (75%) TSCs, respectively, although the populations of responsive cells were not identical. Like TSCs of WT mice, L-amino acids elicited a response in some but not all TSCs when presented with IMP (Table 1; Fig 5). Additionally, IMP presented alone elicited Ca^2+^ responses in 14 of the 24 (58%) responsive TSCs. The magnitude of responses to IMP was 32.6%±6.2 (Mean±SEM). The results from TSCs of T1r3 KO mice lacking one component of the T1r umami receptor show that many of these TSCs are still capable of responding to different L-amino acids. Interestingly, when we compared the percentage of responsive cells between WT and T1r3 KO mice, we found there were significantly fewer responsive cells for the T1r3 KO mice compared to the WT mice (Chi square test; P<0.01) but the proportion of TSCs that responded to each L-amino acid (except for MPG) was significantly larger for T1r3 KO mice (Chi square test; P<0.05). This may reflect the involvement of multiple receptors in the detection of L-amino acids. The absence of one receptor appears to decrease the number of L-amino acid responsive cells. However, the cells that are still capable of detecting an L-amino acids, are utilizing fewer receptor types and consequently making their responses more homogenous. This further supports the hypothesis that although T1r1+T1r3 receptor is involved in L-amino acid transduction, it is not the only receptor involved in L-amino acid taste.

### Receptor(s) other than T1r1+T1r3 may be involved in synergistic responses

**Fig 6:**
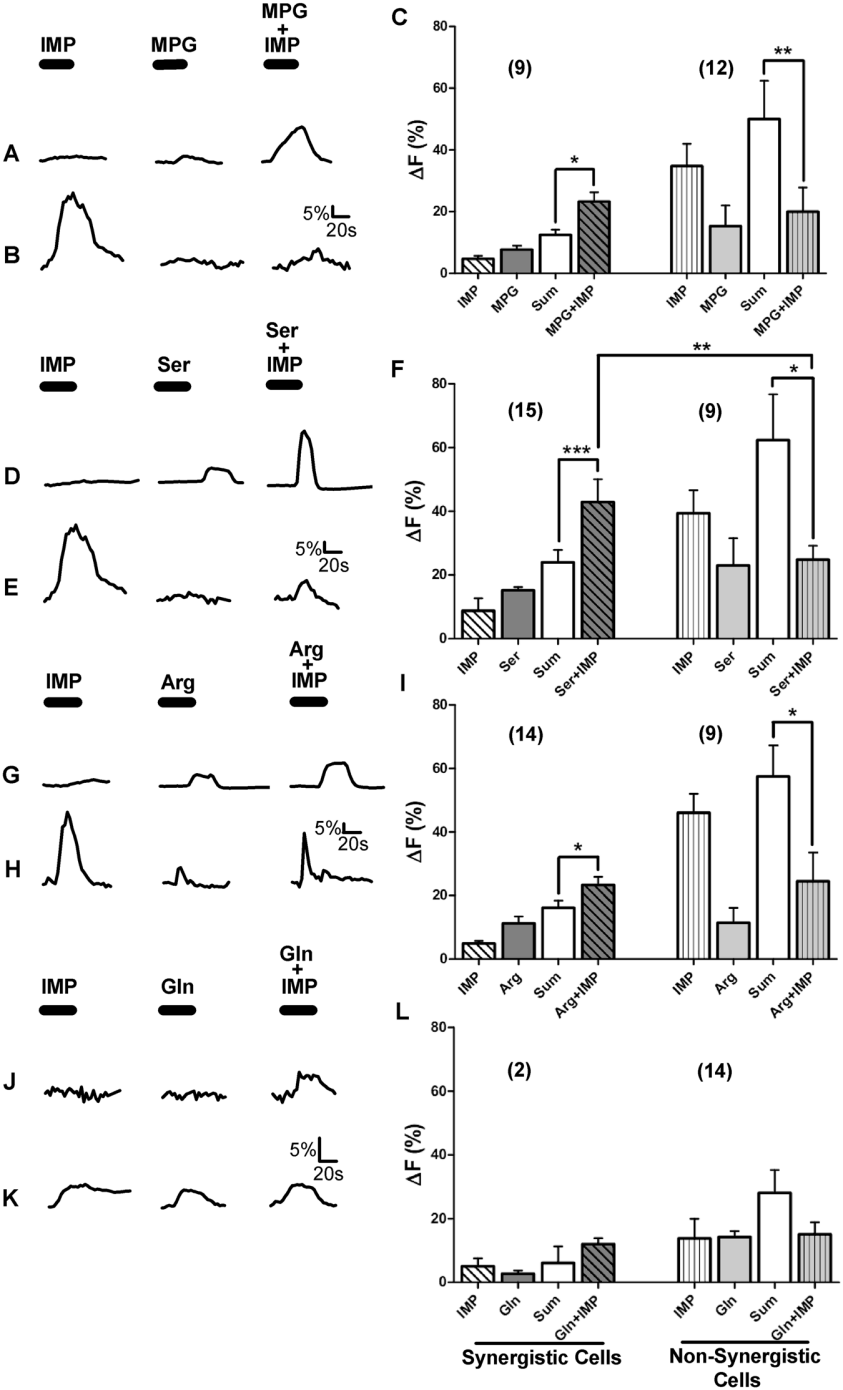
TSCs from T1r3 KO mice can also elicit synergistic responses. Ca^2+^ responses of T1r3 KO TSCs to the four L-amino acid sets are shown. Each L-amino acid set consisted of one of the four L-amino acids (MPG (10mM), Ser (20mM), Arg (10mM), or Gln (10mM), respectively), IMP (1mM), and the MIX of L-amino acid+IMP. [A, D, G, J] Representative TSC responses when the magnitudes of MIX responses were greater than the summation of individual L-amino acid and IMP responses, i.e., the MIX responses were synergistic. [B, E, H, I] Representative TSC responses where the magnitudes of MIX responses were greater than or equal to the response of the L-amino acid or IMP individually, but not greater than the summation of the individual L-amino acid and IMP responses, i.e., the MIX responses were not synergistic. [C, F, I, L] Mean±SEM response for the L-amino acid sets exhibiting synergistic and non-synergistic responses. We were unable to conclude anything about Gln set as only 2 cells responded synergistically to Gln+IMP. Numbers in parenthesis are the number of cells. Mann Whitney test was used for statistical testing. ***P<0.001, **P<0.01, *P<0.05.

We next analyzed responses for each L-amino acid set to determine if TSCs from T1r3 KO mice also showed any synergistic responses. For each L-amino acid set, a subset of MIX responses was greater than the sum of the responses to individual components of the MIX (Table 1; Fig 6). Of 21 MPG+IMP-MIX-responsive cells, 9 (43%) cells exhibited synergistic responses. Likewise for Ser, Arg, and Gln sets 63%, 61%, and 13% of the MIX-responsive cells showed synergistic responses, respectively (Table 1). In addition, similar to WT responses, synergistic MIX responses of the KO mice were significantly greater than the calculated summed responses for MPG, Ser, and Arg set (One way ANOVA; P<0.05) (Fig 6). We further compared the percentage of MIX-responsive cells between WT and T1r3 KO mice. The percentage of cells responding to the MIX for each L-amino acid was not different between WT and T1r3 KO mice (Chi square test; P>0.05). However, for the Ser and Arg sets, the percentages of synergistic cells were significantly greater in T1r3 KO mice (Chi square test; P<0.05). These results further suggest that receptor(s) other than T1r1+T1r3 heterodimer are involved in L-amino acid detection and that other receptors can also elicit synergistic responses.

**Fig 7:**
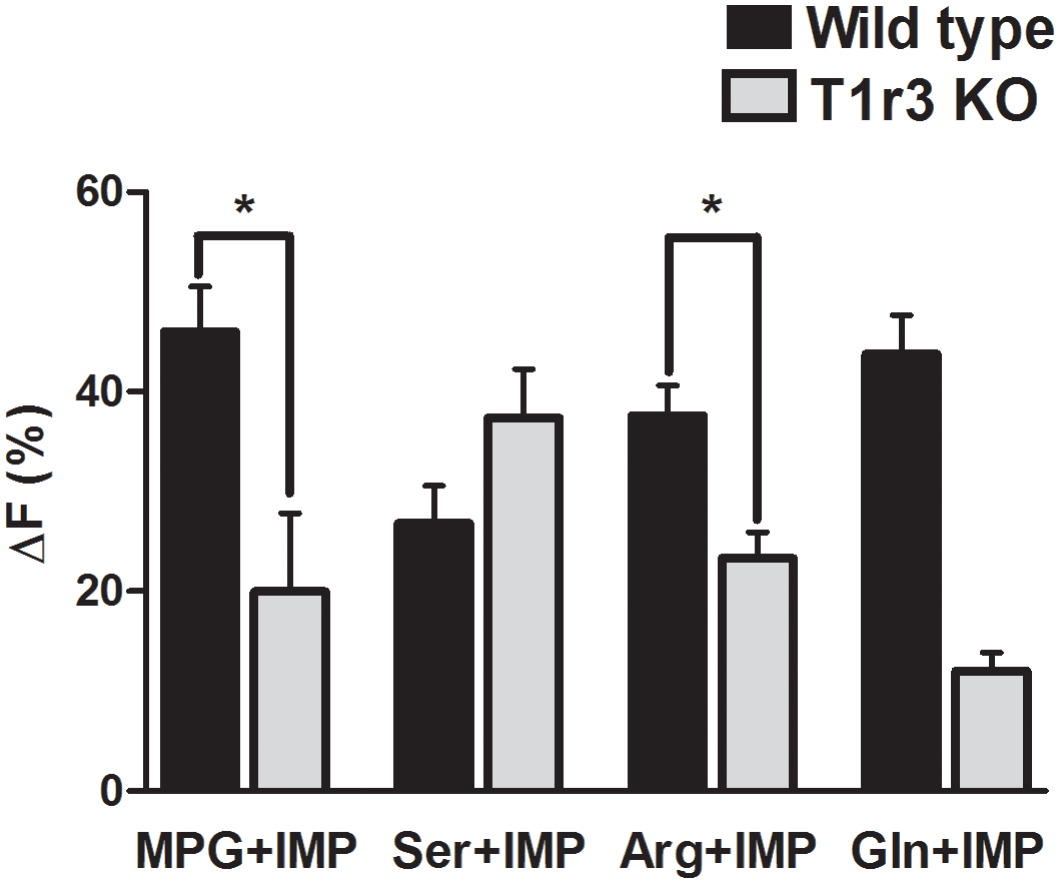
Comparison of amplitude of synergistic responses between WT and T1r3 KO mice. Bars represent Mean±SEM for synergistic responses (i.e., responses to the MIX that were greater than the sum of individual responses) of TSCs from WT and T1r3 KO mice. The amplitude of responses elicited by MPG+IMP and Arg+IMP was significantly smaller in T1r3 KO cells than those of WT cells (Unpaired t-test). *P<0.05.

Clustering of MIX-responsive cells of WT mice to evaluate their responses to individual stimuli showed that almost 100% of the cells that did not respond to one or the other of the individual stimuli i.e., L-amino acid or IMP, responded synergistically to the MIX (Table 2). Interestingly, only three TSCs from T1r3 KO mice exhibited similar response patterns (2 for MPG, 1 for Gln set (Table 3)). Additionally, a more direct comparison of the synergistic responses by TSCs of WT and T1r3 KO mice revealed that the mean amplitude of responses generated by T1r3 KO cells were significantly smaller for MPG+IMP, and Arg+IMP (Unpaired t-test; P<0.05; Fig 7). However, Ser+IMP synergistic responses of T1r3 KO cells were not different from WT cells. Moreover, the mean amplitude of synergistic MIX responses by T1r3 KO cells for the Ser set was significantly greater than non-synergistic MIX responses (Fig 6F). These data suggest that, while the T1r1+T1r3 heterodimer is important for synergistic responses, other receptors also play a role in eliciting synergistic responses.

**Table 3:** Summary of T1r3 KO MIX-responsive synergistic and non-synergistic TSCs.

	**MPG+IMP-MIX-Responsive TSCs**		
Calcium Response	**Synergistic**	**Non-Synergistic**	
YES = **+**; No = **-**	Number of TSCs		**Total**
MPG - / IMP -	1	0	**1 (5)**
MPG - / IMP +	0	4	**4 (19)**
MPG + / IMP -	5	2	**7 (33)**
MPG + / IMP +	3	6	**9 (43)**
**Total**	**9 (43)**	**12 (57)**	**21**
	**Ser+IMP-MIX-Responsive TSCs**		
Calcium Response	**Synergistic**	**Non-Synergistic**	
YES = **+**; No = **-**	Number of TSCs		**Total**
Ser - / IMP -	0	0	**0 (0)**
Ser - / IMP +	0	0	**0 (0)**
Ser + / IMP -	9	1	**10 (42)**
Ser + / IMP +	6	8	**14 (58)**
**Total**	**15 (63)**	**9 (37)**	**24**
	**Arg+IMP-MIX-Responsive TSCs**		
Calcium Response	**Synergistic**	**Non-Synergistic**	
YES = **+**; No = **-**	Number of TSCs		**Total**
Arg - / IMP -	0	0	**0 (0)**
Arg - / IMP +	1	4	**5 (22)**
Arg + / IMP -	9	0	**9 (39)**
Arg + / IMP +	4	5	**9 (39)**
**Total**	**14 (61)**	**9 (39)**	**23**
	**Gln+IMP-MIX-Responsive TSCs**		
Calcium Response	**Synergistic**	**Non-Synergistic**	
YES = **+**; No = **-**	Number of TSCs		**Total**
Gln - / IMP -	1	0	**1 (6)**
Gln - / IMP +	1	0	**1 (6)**
Gln + / IMP -	0	8	**8 (50)**
Gln + / IMP +	0	6	**6 (38)**
**Total**	**2 (12)**	**14 (88)**	**16**

Values are number of cells. Values in parenthesis are percentages. +, response; -, no response to the stimulus.

### TSCs in Clusters

**Fig 8:**
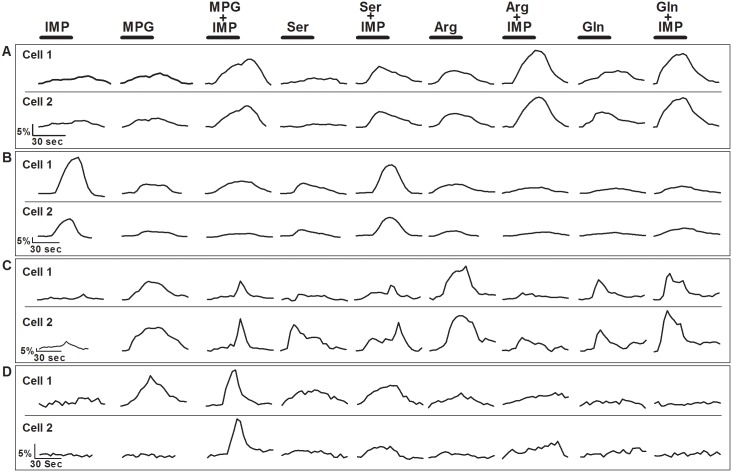
Representative Ca^2+^ responses of adjacent WT TSCs in clusters. Cells were stimulated with 9 different stimuli: IMP (1mM) 4 different L-amino acids (MPG (10mM), Ser (20mM), Arg (10mM), and Gln (10mM)), and L-amino acid+IMP. The bar above each stimulus trace represents the stimulus application time (30 sec). [A] Cells 1 and 2, adjacent TSCs in a cluster, generated Ca^2+^ responses similar in temporal and amplitude to each stimulus, including synergistic responses to all the L-amino acid+IMP MIXes. [B] These cells were adjacent TSCs in a cluster, exhibited similar non-synergistic Ca^2+^ responses to all the L-amino acid+IMP MIXes, but the response amplitudes of cell 2 was 50% of cell 1. [C] Cells 1 and 2 were adjacent TSCs in a cluster that responded to the same stimuli. However, one cell responded synergistically to the MIXes whereas the other cell responded non-synergistically. [D] Cells 1 and 2 were adjacent TSCs. Each cell exhibited different response patterns to the array of test stimuli.

In our experiments isolated TSCs and TSCs in clusters were examined. TSCs in clusters are more stable, and stayed healthy for longer periods, thus allowing longer imaging time. One drawback of using clusters of cells, however, can be indirect activation of some TSCs in direct contact with another cell. There are two possible models through which an L-amino acid taste stimulus can activate a second-messenger-dependent (inositol triphosphate, IP_3_) calcium wave that propagates between adjacent TSCs: (1) IP_3_ may traverses through gap junctions and initiates the release of intracellular calcium stores in neighboring Type II or III cells (as found in different cellular systems) [50], (2) a Type II cell releases ATP that acts on a Type III (presynaptic) cell to increase intracellular Ca^2+^ which e.g., could lead to release of neurotransmitter stored in vesicles [47, 51–53]. In either case, adjacent cells could generate similar response patterns within our imaging procedures. To identify cells that might be responding indirectly, we analyzed the response patterns of cells adjacent to each other. Of the 170 cells, 19% of the cells were close enough together for one of the cells to potentially be activated by another. Some of the adjacent cells in a cluster generated Ca^2+^ response patterns with comparable time courses and amplitudes. For example, cells 1 and 2 in Fig 8A responded to all 9 stimuli with very similar increases in intracellular Ca^2+^. Furthermore, both cells generated synergistic responses to all four L-amino acid+IMP MIXes (Fig 8A). Similarly, cell 1 and cell 2 in Fig 8B generated comparable Ca^2+^ response patterns to all 9 stimuli but in this case the response amplitude of cell 2 was around 50% of cell 1, and their responses were non-synergistic to all the four L-amino acid+IMP MIXes. In another instance, while one cell generated a synergistic response to the L-amino acids+IMP MIXes, the responses of the adjacent cell were non-synergistic (Fig 8C). Thus, even though adjacent cells might appear to have similar response patterns to a stimulus, they often did not generate responses with the same temporal and intensity characteristics. In other cases, adjacent cells did not have comparable response patterns. For example, Fig 8D shows Ca^2+^ responses of a pair of cells that had quite different response patterns to the 9 stimulus compounds. Conversely, none of the response patterns of adjacent cells were unique when compared to those of isolated cells, but rather they generated responses that were generally indistinguishable from isolated TSCs. When we eliminated 50% of these adjacent cells (assuming half of the cells responded to L-amino acid stimuli, and the rest demonstrated a Ca^2+^ increase due to indirect activation) from their respective data sets, there was a small reduction in the number of cells per group but no change in the overall findings from our experiments. Similar observations were made for TSCs of T1r3 KO mice.

## Discussion

Although much is known about the receptor systems and transduction mechanisms involved in the detection of L-glutamate, the prototypical umami L-amino acid, the mechanisms for detecting other L-amino acids are not well understood. In general, detection of L-amino acids by the taste system has been linked closely to detection of umami stimuli through a presumed common taste receptor and through interactions with 5’-ribonucleotides. The impetus for this connection was strengthened considerably by the discovery that most L-amino acids appear to be able to activate T1r1+T1r3 receptors in HEK cells, especially when they were mixed with IMP, and by a number of subsequent studies [23–25]. There is growing evidence, however, that other receptors may contribute to umami taste sensations, including taste-mGluR4, taste-mGluR1 and possibly others [20, 29, 30, 54, 55]. If a single receptor is responsible for detecting all L-amino acids, then they should induce responses in the same TSCs and elicit the same or similar taste qualities, including synergistic responses when they are mixed with 5’-ribonucleotides. However, conditioned taste aversion and discrimination studies in rats and mice have shown that L-amino acids do not elicit the same taste qualities [33, 55, 56]. Similarly, psychophysical studies have shown that humans perceive Ser and Gln as sweet at low concentrations and umami at high concentrations but they perceive Arg as bitter [34, 35]. The results of these experiments appear to be more in consistent with the hypothesis that the sensations elicited by each L-amino acid may be the product of the combined contributions of multiple L-amino acid receptors rather than a single receptor.

To more directly evaluate this hypothesis, Ca^2+^ imaging of isolated mouse TSCs and taste cell clusters were performed with a panel of four L-amino acids (MPG, Ser, Arg, and Gln). Our aim was to determine if single TSCs are responsive to all or a subset of L-amino acids and whether these cells showed evidence of synergy when mixed with IMP. We observed a wide range of response patterns of single TSCs tested in isolation or in clusters with all 9 stimuli but only a few TSCs responded to all 9 stimuli (10 out of 170 cells; 6%). Mixing IMP with L-amino acids elicited synergy for all 4 L-amino acids tested, but not every MIX-responsive cells responded synergistically. In addition, TSCs from T1r3 KO mice showed response patterns comparable to those of WT mice.

Since we bath applied stimuli, some responses may be due to activation of glutamate receptors expressed in the basolateral membrane of the cells. Several neurotransmitters have been proposed to function in the taste buds, including glutamate, serotonin, gamma amino butyric acid, norepinephrine, acetylcholine, ATP, CCK, and neuropeptide Y [57–74], but only serotonin, norepinephrine, and ATP have been unambiguously identified and shown to be released in response to stimulation [60, 61, 64–66]. Studies suggesting glutamate as a potential neurotransmitter are mainly based upon the expression of ionotropic and metabotropic glutamate receptors in the lingual tissue, including taste buds, and the expression of glutamate transporter GLAST in Type I taste cells [20, 21, 47, 58, 67, 72, 75]. Vandenbeuch et al. [72] found vesicular glutamate transporters, VGLUT1 and 2, expressed in the afferent nerve fibers, but not in taste bud cells. Thus glutamate may be released by afferent nerve fibers, and may modulate taste function [58, 62, 72, 75]. However, to date there is no report that directly shows the release of glutamate in taste buds, or any modulatory function of glutamate in the taste buds. Nevertheless, we realize this may be a limitation of our protocol, and that there may be glutamate receptors at the basolateral end of the TSCs. If so, some of the responses will not be normal taste responses.

### Multiple receptors and/or transduction mechanisms are involved in L-amino acids and IMP taste

In our experiments, TSCs of WT mice often responded to more than one L-amino acid, but not all L-amino acids elicited a response in the same TSC (Table 1; Fig 1). Some but not all L-amino acid responsive cells responded to glutamate, the prototypical umami taste stimulus. When TSCs were stimulated with the MIX of an L-amino acid and IMP, a diverse array of response patterns were found. For example, 1) the MIX for each L-amino acid elicited a response in some TSCs, 2) cells that responded to the individual components of a MIX did not always respond when the MIX was applied, and 3) a subset of MIX-responsive TSCs did not respond to the individual components of the MIX (Table 2; Fig 2). Prior recording studies of the chorda tympani (CT) and glossopharyngeal (GL) nerve fibers of rats and mice have shown that 5’-ribonucleotides or glutamate alone can elicit measurable responses. Although many nerve fibers responded to IMP, GMP, and glutamate, some responded to only 5’-ribonucleotides or glutamate. In addition, only a subset of fiber responses to a glutamate+5’ ribonucleotide-MIX was synergistic [44, 76–82]. Similar response patterns were also seen in patch clamp recordings and Ca^2+^ imaging experiments. Lin et al. [44] using taste cells isolated from rat fungiform papillae showed that a subset of TSCs responded to glutamate or GMP alone, and only a subset of glutamate+GMP-MIX-responsive cells showed synergy. Further, behavioral discrimination studies have shown that rats could positivity distinguish between MSG and IMP or GMP, suggesting that MSG and these 5’ ribonucleotides possess at least some unique taste qualities [83]. Our Ca^2+^ imaging results are consistent with these previous studies and suggest that TSCs may respond to IMP and the L-amino acids with different receptors and/or transduction pathways.

Our findings with isolated or clustered TSCs did not recapitulate response trends seen withT1r1+T1r3 heterodimer expressing HEK cells [24]. These HEK cells showed no Ca^2+^ increase when stimulated with the prototypical umami compound MSG (50mM). However, in our study 46% of the responsive TSCs responded to 10mM MPG presented alone. These differences in cellular responses might be due to the experimental model (*in vivo* versus *in vitro*) in which the receptor heterodimer is expressed, but it is also plausible that receptors other than T1r1+T1r3 are involved in L-amino acids and IMP detection.

### In posterior tongue, receptor(s) other than T1r1+T1r3 can generate synergistic responses

Comparison of TSCs from WT and T1r3 KO mice revealed some very interesting findings, especially when IMP was a part of the stimulus solution. Synergy between 5’-ribonucleotides and glutamate is a defining characteristic of umami taste. In HEK cells expressing T1r1+T1r3, several L-amino acids exhibited this synergistic characteristic in the presence of IMP [24], often responding to an L-amino acid only when IMP was present. A previous study reported that some TSCs responded synergistically to glutamate+IMP but had only small or no responses to glutamate or GMP alone [44]. Our analysis of the WT TSC responses to the 9 stimuli (IMP, 4 L-amino acids, with and without IMP) revealed an interesting cluster of 40 (24%) cells which responded to L-amino acids only in presence of IMP, and showed little or no response to IMP alone. We also identified a subset of synergistic WT cells that had no detectable response to the individual components of the MIX for all four L-amino acids tested. This resulted in a noticeable increase in the number of cells responsive to L-amino acids in presence of IMP (Table 1). Interestingly, we did not find any such response patterns for TSCs of T1r3 KO mice. In the WT experiments, all 4 L-amino acid sets elicited synergistic MIX responses that had significantly higher peak amplitudes than non-synergistic MIX responses (Fig 2C, 2F, 2I and 2L). In contrast, T1r3 KO cells responded synergistically to the MIXes but the responses to 2 of the 3 sets were not significantly larger than the non-synergistic MIX responses (Ser+IMP was the exception) (Fig 6C, 6F and 6I). We were unable to conclude anything about the Gln set, as only 2 cells responded synergistically to Gln+IMP. These findings suggest that there is a different response mechanism that is dependent upon the L-amino acid ligand. This conclusion is further supported by synergistic responses of T1r3 KO cells. The responses of these cells to MPG+IMP and Arg+IMP-MIXes were significantly smaller than responses of WT cells (Fig 7), whereas responses to Ser+IMP were not different from WT TSCs (Fig 7). Collectively, these results suggest that although the T1r1+T1r3 heterodimer plays an important role in generating synergistic responses to these L-amino acids, it is not the only receptor capable of eliciting a synergistic response.

The characteristics of single fiber and whole-nerve responses to stimulation by L-amino acids vary considerably between CT and GL nerves [78, 80]. Ninomiya and colleagues identified taste fibers in the mouse GL nerve that responded to umami compound MSG, including fibers showing synergistic responses to MSG in the presence of 0.5mM GMP [84, 85]. The importance of the GL nerve in umami taste was further established when Ninomiya and Funakoshi showed that mice with bilateral section of the GL nerve could not discriminate between MSG and NaCl [84, 85]. Moreover, the posterior part of the tongue is more sensitive to umami than the anterior tongue [86, 87]. On the other hand, whole nerve responses to umami are much greater in the CT than in the GL and synergy is detectable in the response of the CT but not the GL [88]. In addition, the sweet taste inhibiting peptide gurmarin inhibited the umami signal and synergistic responses preferentially in the CT of C57BL/6J mice, but had no detectible effect on GL nerve recordings. Thus there may be a different set and/or proportion of receptors that elicit umami taste in the posterior portion of the tongue. Even though whole nerve studies found minimal synergistic responses from the posterior portion of the tongue, in this study we found a heterogeneous group of cells in circumvallate and foliate taste buds that showed synergistic responses to L-amino acids and IMP stimuli.

Recently the Venus fly trap domain of the T1r1 subunit of the T1r1+T1r3 heterodimer was proposed to be critical for umami synergism [89, 90]. We found an unexpectedly high proportion of TSCs from T1r3 KO mice, like those of WT mice, responded not only to L-glutamate but also to other L-amino acids, with or without IMP. Some of these KO cells also exhibited synergistic responses when L-amino acids were mixed with IMP (Table 1; Fig 2). The combination of these findings suggests that there is likely another receptor at least in the posterior region of the tongue that is capable of eliciting synergistic responses. In circumvallate papillae, the majority of the T1r3 expressing cells also express T1r1, but only around 50% of the T1r1 expressing cells co-express T1r3 [91]. Thus it is possible that T1r1 may homodimerize or form heterodimers with other receptors, but at present there is little evidence supporting this possibility. Additionally, the expression of the T1r1+T1r3 heterodimer is not uniform throughout the different taste papillae and is lower in the posterior portion of the tongue [91–96]. Taste-mGluR4 has been proposed to be an alternate receptor involved in umami taste. The action of L-AP4, an agonist of taste-mGluR4, has been shown to be enhanced in the presence of IMP [96, 97]. Thus, taste-mGluR4 alone or with other receptor complexes may be involved in mediating synergistic responses.

### IMP alone elicits responses in TSCs

Our finding that IMP alone can elicit a response in a large number of TSCs was surprising since Lin et al. [44] reported finding far fewer fungiform TSCs in the rat that responded to GMP. This might be due to the difference in the species and the papillae from which the cells were isolated. However, the receptor involved in the detection of 5’-ribonucleotides remains unclear. An IMP binding site has been proposed to be located in the N-terminal domain of the T1r1 subunit [90], but the lack of IMP-induced Ca^2+^ responses in HEK cells expressing T1r1 or the heterodimer T1r1+T1r3 raises questions about its role as an IMP receptor. In our study, there was no difference between WT and T1r3 KO mice in the percentage of IMP responding cells. IMP responses by TSCs of T1r3 KO mice further support the hypothesis that receptor(s) other than the T1r1+T1r3 heterodimer can be activated by IMP. Studies with GPCRs and their agonists have shown that a single compound can act as either an agonist or an allosteric modulator, depending on receptor binding [98–100]. It is possible that IMP might act as either an agonist where IMP itself can elicit significant Ca^2+^ responses without enhancing the response to another substance such as an L-amino acid, or as an allosteric modulator where it facilitates L-amino acid responses such as when IMP induces a synergistic response. Thus, it is possible that synergistic responses elicited by L-amino acids in presence of IMP are mediated primarily but not exclusively by the T1r1+T1r3 heterodimer, where L-amino acids act as umami compounds and IMP acts as an allosteric modulator. Other TSCs may have receptors that mediate non-synergistic responses to individual L-amino acids or IMP.

Although the specific receptors involved in detection of L-amino acids and IMP cannot be identified from these data, there may be several possibilities. Besides the T1r1+T1r3 heterodimer, it seems likely that mGluR receptors, including brain and truncated taste versions of mGluR4 and mGluR1 and possibly mGluR2 and mGluR3, may be potential candidates [20, 29–32, 54, 55]. Recently, the Ca^2+^ sensor CaSR and the class C GPCR, GPRC6A, which can also detect L-amino acids, were localized to Type I and III taste cells [101, 102]. However, whether these Ca^2+^ sensors can generate synergistic responses is yet to be determined. One very intriguing possibility is that taste-mGluR4 or some other receptor may form a complex with each other or with T1rs that responds to L-amino acids as well as generate synergistic responses. However, further investigation is needed to examine this hypothesis.

## Conclusions

In summary, we report for the first time the response patterns of single TSCs to IMP and four L-amino acids (from different classes) with and without IMP. Our data strongly suggest that, in addition to T1r1+T1r3, one or more receptor(s) other than T1rs contribute to the tastes of IMP and L-amino acids, as well as to synergistic interactions between IMP and L-amino acids. In particular, using Ca^2+^ imaging we showed that response patterns elicited by L-amino acids varied significantly across isolated TSCs. IMP and all four L-amino acids elicited Ca^2+^ responses in TSCs, although each cell typically responded to multiple, but not all L-amino acids. Moreover, in the presence of IMP, L-amino acids other than glutamate were able to elicit synergistic responses. Along with its role in synergism, IMP alone was capable of eliciting Ca^2+^ responses in TSCs of WT and T1r3 KO mice. We also found that TSCs from T1r3 KO mice can respond to L-amino acids and at least some are capable of synergistic responses in the presence of IMP. These results suggest that multiple receptors are involved in IMP and L-amino acid detection, as well as in generating synergistic responses.

## References

[1] Beauchamp GK, Bertino M, Engelman K. Modification of salt taste. Annals of internal medicine. 1983;98(5 Pt 2):763–9. 6847015 10.7326/0003-4819-98-5-763

[2] Bertino M, Beauchamp GK, Engelman K. Long-term reduction in dietary sodium alters the taste of salt. The American journal of clinical nutrition. 1982;36(6):1134–44. 7148734 10.1093/ajcn/36.6.1134

[3] DeMet E, Stein MK, Tran C, Chicz-DeMet A, Sangdahl C, Nelson J. Caffeine taste test for panic disorder: adenosine receptor supersensitivity. Psychiatry research. 1989;30(3):231–42. 2616690 10.1016/0165-1781(89)90014-0

[4] Dess NK, Edelheit D. The bitter with the sweet: the taste/stress/temperament nexus. Biological psychology. 1998;48(2):103–19. 9700013 10.1016/s0301-0511(98)00014-3

[5] Fabbi F. Gustatory sense modifications in diabetes. Archiv fur Ohren-, Nasen- und Kehlkopfheilkunde, vereinigt mit Zeitschrift fur Hals-, Nasen- und Ohrenheilkunde. 1954;164(6):543–6. 13189449 10.1007/BF02105163

[6] Le Floch JP, Le Lievre G, Sadoun J, Perlemuter L, Peynegre R, Hazard J. Taste impairment and related factors in type I diabetes mellitus. Diabetes care. 1989;12(3):173–8. 2702907 10.2337/diacare.12.3.173

[7] Maliphol AB, Garth DJ, Medler KF. Diet-induced obesity reduces the responsiveness of the peripheral taste receptor cells. PloS one. 2013;8(11):e79403 10.1371/journal.pone.0079403 24236129 PMC3827352

[8] Mattes RD. Salt taste and hypertension: a critical review of the literature. Journal of chronic diseases. 1984;37(3):195–208. 6365942 10.1016/0021-9681(84)90147-4

[9] Schiffman S. Changes in taste and smell: drug interactions and food preferences. Nutrition reviews. 1994;52(8 Pt 2):S11–4. 7970295 10.1111/j.1753-4887.1994.tb01439.x

[10] Steiner JE, Rosenthal-Zifroni A, Edelstein EL. Taste perception in depressive illness. The Israel annals of psychiatry and related disciplines. 1969;7(2):223–32. 5274360

[11] Wagner A, Aizenstein H, Mazurkewicz L, Fudge J, Frank GK, Putnam K, et al Altered insula response to taste stimuli in individuals recovered from restricting-type anorexia nervosa. Neuropsychopharmacology: official publication of the American College of Neuropsychopharmacology. 2008;33(3):513–23. 17487228 10.1038/sj.npp.1301443

[12] Maga JA. Flavor potentiators. Critical reviews in food science and nutrition. 1983;18(3):231–312. 6137322 10.1080/10408398309527364

[13] Yamaguchi S, Ninomiya K. Umami and food palatability. The Journal of nutrition. 2000;130(4S Suppl):921S–6S. 10736353 10.1093/jn/130.4.921S

[14] Bellisle F, Monneuse MO, Chabert M, Larue-Achagiotis C, Lanteaume MT, Louis-Sylvestre J. Monosodium glutamate as a palatability enhancer in the European diet. Physiology & behavior. 1991;49(5):869–73. 1886949 10.1016/0031-9384(91)90196-u

[15] Bellisle F. Glutamate and the UMAMI taste: sensory, metabolic, nutritional and behavioural considerations. A review of the literature published in the last 10 years. Neuroscience and biobehavioral reviews. 1999;23(3):423–38. 9989429 10.1016/s0149-7634(98)00043-8

[16] Bellisle F. Experimental studies of food choices and palatability responses in European subjects exposed to the Umami taste. Asia Pacific journal of clinical nutrition. 2008;17 Suppl 1:376–9. 18296383

[17] Schiffman SS. Intensification of sensory properties of foods for the elderly. The Journal of nutrition. 2000;130(4S Suppl):927S–30S. 10736354 10.1093/jn/130.4.927S

[18] Toyama K, Tomoe M, Inoue Y, Sanbe A, Yamamoto S. A possible application of monosodium glutamate to nutritional care for elderly people. Biological & pharmaceutical bulletin. 2008;31(10):1852–4. 18827342 10.1248/bpb.31.1852

[19] Yamamoto S, Tomoe M, Toyama K, Kawai M, Uneyama H. Can dietary supplementation of monosodium glutamate improve the health of the elderly? The American journal of clinical nutrition. 2009;90(3):844S–9S. 10.3945/ajcn.2009.27462X 19571225

[20] Chaudhari N, Yang H, Lamp C, Delay E, Cartford C, Than T, et al The taste of monosodium glutamate: membrane receptors in taste buds. The Journal of neuroscience: the official journal of the Society for Neuroscience. 1996;16(12):3817–26. 8656276 10.1523/JNEUROSCI.16-12-03817.1996 PMC6578609

[21] Chaudhari N, Landin AM, Roper SD. A metabotropic glutamate receptor variant functions as a taste receptor. Nature neuroscience. 2000;3(2):113–9. 10649565 10.1038/72053

[22] Damak S, Rong M, Yasumatsu K, Kokrashvili Z, Varadarajan V, Zou S, et al Detection of sweet and umami taste in the absence of taste receptor T1r3. Science. 2003;301(5634):850–3. 12869700 10.1126/science.1087155

[23] Li X, Staszewski L, Xu H, Durick K, Zoller M, Adler E. Human receptors for sweet and umami taste. Proceedings of the National Academy of Sciences of the United States of America. 2002;99(7):4692–6. 11917125 10.1073/pnas.072090199 PMC123709

[24] Nelson G, Chandrashekar J, Hoon MA, Feng L, Zhao G, Ryba NJ, et al An amino-acid taste receptor. Nature. 2002;416(6877):199–202. 11894099 10.1038/nature726

[25] Zhao GQ, Zhang Y, Hoon MA, Chandrashekar J, Erlenbach I, Ryba NJ, et al The receptors for mammalian sweet and umami taste. Cell. 2003;115(3):255–66. 14636554 10.1016/s0092-8674(03)00844-4

[26] Delay ER, Hernandez NP, Bromley K, Margolskee RF. Sucrose and monosodium glutamate taste thresholds and discrimination ability of T1R3 knockout mice. Chemical senses. 2006;31(4):351–7. 16495435 10.1093/chemse/bjj039

[27] Kusuhara Y, Yoshida R, Ohkuri T, Yasumatsu K, Voigt A, Hubner S, et al Taste responses in mice lacking taste receptor subunit T1R1. The Journal of physiology. 2013;591(Pt 7):1967–85. 10.1113/jphysiol.2012.236604 23339178 PMC3624863

[28] Nakashima K, Eddy MC, Katsukawa H, Delay ER, Ninomiya Y. Behavioral responses to glutamate receptor agonists and antagonists implicate the involvement of brain-expressed mGluR4 and mGluR1 in taste transduction for umami in mice. Physiology & behavior. 2012;105(3):709–19. 22008743 10.1016/j.physbeh.2011.09.028

[29] San Gabriel A, Uneyama H, Yoshie S, Torii K. Cloning and characterization of a novel mGluR1 variant from vallate papillae that functions as a receptor for L-glutamate stimuli. Chemical senses. 2005;30 Suppl 1:i25–6. 15738140 10.1093/chemse/bjh095

[30] San Gabriel A, Maekawa T, Uneyama H, Torii K. Metabotropic glutamate receptor type 1 in taste tissue. The American journal of clinical nutrition. 2009;90(3):743S–6S. 10.3945/ajcn.2009.27462I 19571209

[31] Toyono T, Kataoka S, Seta Y, Shigemoto R, Toyoshima K. Expression of group II metabotropic glutamate receptors in rat gustatory papillae. Cell and tissue research. 2007;328(1):57–63. 17216195 10.1007/s00441-006-0351-9

[32] Eschle BK, Eddy MC, Delay ER. Antagonism of metabotropic glutamate receptor 4 receptors by (RS)-alpha-cyclopropyl-4-phosphonophenylglycine alters the taste of amino acids in rats. Neuroscience. 2009;163(4):1292–301. 10.1016/j.neuroscience.2009.07.035 19631258

[33] Delay ER, Mitzelfelt JD, Westburg AM, Gross N, Duran BL, Eschle BK. Comparison of L-monosodium glutamate and L-amino acid taste in rats. Neuroscience. 2007;148(1):266–78. 17629624 10.1016/j.neuroscience.2007.05.045

[34] Kawai M, Sekine-Hayakawa Y, Okiyama A, Ninomiya Y. Gustatory sensation of (L)- and (D)-amino acids in humans. Amino acids. 2012;43(6):2349–58. 10.1007/s00726-012-1315-x 22588481

[35] Schiffman SS, Sennewald K, Gagnon J. Comparison of taste qualities and thresholds of D- and L-amino acids. Physiology & behavior. 1981;27(1):51–9. 7267802 10.1016/0031-9384(81)90298-5

[36] Maruyama Y, Pereira E, Margolskee RF, Chaudhari N, Roper SD. Umami responses in mouse taste cells indicate more than one receptor. The Journal of neuroscience: the official journal of the Society for Neuroscience. 2006;26(8):2227–34. 16495449 10.1523/JNEUROSCI.4329-05.2006 PMC3717266

[37] Damak S, Mosinger B, Margolskee RF. Transsynaptic transport of wheat germ agglutinin expressed in a subset of type II taste cells of transgenic mice. BMC neuroscience. 2008;9:96 10.1186/1471-2202-9-96 18831764 PMC2571104

[38] He W, Yasumatsu K, Varadarajan V, Yamada A, Lem J, Ninomiya Y, et al Umami taste responses are mediated by alpha-transducin and alpha-gustducin. The Journal of neuroscience: the official journal of the Society for Neuroscience. 2004;24(35):7674–80. 15342734 10.1523/JNEUROSCI.2441-04.2004 PMC6729622

[39] Sako N, Yamamoto T. Analyses of taste nerve responses with special reference to possible receptor mechanisms of umami taste in the rat. Neuroscience letters. 1999;261(1–2):109–12. 10081939 10.1016/s0304-3940(99)00019-1

[40] Pritchard TC, Scott TR. Amino acids as taste stimuli. I. Neural and behavioral attributes. Brain research. 1982;253(1–2):81–92. 7150976 10.1016/0006-8993(82)90675-8

[41] Yoshii K, Yokouchi C, Kurihara K. Synergistic effects of 5'-nucleotides on rat taste responses to various amino acids. Brain research. 1986;367(1–2):45–51. 3008926 10.1016/0006-8993(86)91577-5

[42] Nagarajan S, Kellogg MS, DuBois GE, Hellekant G. Understanding the mechanism of sweet taste: synthesis of ultrapotent guanidinoacetic acid photoaffinity labeling reagents. Journal of medicinal chemistry. 1996;39(21):4167–72. 8863794 10.1021/jm960349q

[43] Behe P, DeSimone JA, Avenet P, Lindemann B. Membrane currents in taste cells of the rat fungiform papilla. Evidence for two types of Ca currents and inhibition of K currents by saccharin. The Journal of general physiology. 1990;96(5):1061–84. 2280253 10.1085/jgp.96.5.1061 PMC2229027

[44] Gilbertson TA, Fontenot DT, Liu L, Zhang H, Monroe WT. Fatty acid modulation of K+ channels in taste receptor cells: gustatory cues for dietary fat. The American journal of physiology. 1997;272(4 Pt 1):C1203–10. 9142845 10.1152/ajpcell.1997.272.4.C1203

[45] Lin W, Ogura T, Kinnamon SC. Responses to di-sodium guanosine 5'-monophosphate and monosodium L-glutamate in taste receptor cells of rat fungiform papillae. Journal of neurophysiology. 2003;89(3):1434–9. 12626621 10.1152/jn.00994.2002

[46] Tomchik SM, Berg S, Kim JW, Chaudhari N, Roper SD. Breadth of tuning and taste coding in mammalian taste buds. The Journal of neuroscience: the official journal of the Society for Neuroscience. 2007;27(40):10840–8. 17913917 10.1523/JNEUROSCI.1863-07.2007 PMC3717408

[47] Dando R, Roper SD. Cell-to-cell communication in intact taste buds through ATP signalling from pannexin 1 gap junction hemichannels. The Journal of physiology. 2009;587(Pt 24):5899–906. 10.1113/jphysiol.2009.180083 19884319 PMC2808547

[48] Sato T, Beidler LM. Broad tuning of rat taste cells for four basic taste stimuli. Chemical senses. 1997;22(3):287–93. 9218141 10.1093/chemse/22.3.287

[49] Gilbertson TA, Boughter JD Jr, Zhang H, Smith DV. Distribution of gustatory sensitivities in rat taste cells: whole-cell responses to apical chemical stimulation. The Journal of neuroscience: the official journal of the Society for Neuroscience. 2001;21(13):4931–41. 11425921 10.1523/JNEUROSCI.21-13-04931.2001 PMC6762376

[50] Boitano S, Dirksen ER, Sanderson MJ. Intercellular propagation of calcium waves mediated by inositol trisphosphate. Science. 1992;258(5080):292–5. 1411526 10.1126/science.1411526

[51] Huang YJ, Maruyama Y, Dvoryanchikov G, Pereira E, Chaudhari N, Roper SD. The role of pannexin 1 hemichannels in ATP release and cell-cell communication in mouse taste buds. Proceedings of the National Academy of Sciences of the United States of America. 2007;104(15):6436–41. 17389364 10.1073/pnas.0611280104 PMC1851090

[52] Romanov RA, Rogachevskaja OA, Bystrova MF, Jiang P, Margolskee RF, Kolesnikov SS. Afferent neurotransmission mediated by hemichannels in mammalian taste cells. The EMBO journal. 2007;26(3):657–67. 17235286 10.1038/sj.emboj.7601526 PMC1794384

[53] Taruno A, Vingtdeux V, Ohmoto M, Ma Z, Dvoryanchikov G, Li A, et al CALHM1 ion channel mediates purinergic neurotransmission of sweet, bitter and umami tastes. Nature. 2013;495(7440):223–6. 10.1038/nature11906 23467090 PMC3600154

[54] Toyono T, Seta Y, Kataoka S, Harada H, Morotomi T, Kawano S, et al Expression of the metabotropic glutamate receptor, mGluR4a, in the taste hairs of taste buds in rat gustatory papillae. Archives of histology and cytology. 2002;65(1):91–6. 12002614 10.1679/aohc.65.91

[55] Toyono T, Seta Y, Kataoka S, Kawano S, Shigemoto R, Toyoshima K. Expression of metabotropic glutamate receptor group I in rat gustatory papillae. Cell and tissue research. 2003;313(1):29–35. 12898387 10.1007/s00441-003-0740-2

[56] Kasahara T, Iwasaki K, Sato M. Taste effectiveness of some D- and L-amino acids in mice. Physiology & behavior. 1987;39(5):619–24. 3588708 10.1016/0031-9384(87)90162-4

[57] Cao Y, Zhao FL, Kolli T, Hivley R, Herness S. GABA expression in the mammalian taste bud functions as a route of inhibitory cell-to-cell communication. Proceedings of the National Academy of Sciences of the United States of America. 2009;106(10):4006–11. 10.1073/pnas.0808672106 19223578 PMC2656195

[58] Caicedo A, Jafri MS, Roper SD. In situ Ca2+ imaging reveals neurotransmitter receptors for glutamate in taste receptor cells. The Journal of neuroscience: the official journal of the Society for Neuroscience. 2000;20(21):7978–85. 11050118 10.1523/JNEUROSCI.20-21-07978.2000 PMC6772752

[59] Dvoryanchikov G, Huang YA, Barro-Soria R, Chaudhari N, Roper SD. GABA, its receptors, and GABAergic inhibition in mouse taste buds. The Journal of neuroscience: the official journal of the Society for Neuroscience. 2011;31(15):5782–91. 10.1523/JNEUROSCI.5559-10.2011 21490220 PMC3320853

[60] Finger TE, Danilova V, Barrows J, Bartel DL, Vigers AJ, Stone L, et al ATP signaling is crucial for communication from taste buds to gustatory nerves. Science. 2005;310(5753):1495–9. 16322458 10.1126/science.1118435

[61] Huang YA, Dando R, Roper SD. Autocrine and paracrine roles for ATP and serotonin in mouse taste buds. The Journal of neuroscience: the official journal of the Society for Neuroscience. 2009;29(44):13909–18. 10.1523/JNEUROSCI.2351-09.2009 19890001 PMC2821712

[62] Huang YA, Grant J, Roper S. Glutamate may be an efferent transmitter that elicits inhibition in mouse taste buds. PloS one. 2012;7(1):e30662 10.1371/journal.pone.0030662 22292013 PMC3266908

[63] Huang YA, Pereira E, Roper SD. Acid stimulation (sour taste) elicits GABA and serotonin release from mouse taste cells. PloS one. 2011;6(10):e25471 10.1371/journal.pone.0025471 22028776 PMC3197584

[64] Huang YJ, Maruyama Y, Lu KS, Pereira E, Plonsky I, Baur JE, et al Mouse taste buds use serotonin as a neurotransmitter. The Journal of neuroscience: the official journal of the Society for Neuroscience. 2005;25(4):843–7. 15673664 10.1523/JNEUROSCI.4446-04.2005 PMC6725637

[65] Huang YJ, Maruyama Y, Lu KS, Pereira E, Roper SD. Mouse taste buds release serotonin in response to taste stimuli. Chemical senses. 2005;30 Suppl 1:i39–40. 15738184 10.1093/chemse/bjh102

[66] Kaya N, Shen T, Lu SG, Zhao FL, Herness S. A paracrine signaling role for serotonin in rat taste buds: expression and localization of serotonin receptor subtypes. American journal of physiology: Regulatory, integrative and comparative physiology. 2004;286(4):R649–58. 14715493 10.1152/ajpregu.00572.2003

[67] Lin W, Kinnamon SC. Physiological evidence for ionotropic and metabotropic glutamate receptors in rat taste cells. Journal of neurophysiology. 1999;82(5):2061–9. 10561387 10.1152/jn.1999.82.5.2061

[68] Lu SG, Zhao FL, Herness S. Physiological phenotyping of cholecystokinin-responsive rat taste receptor cells. Neuroscience letters. 2003;351(3):157–60. 14623130 10.1016/j.neulet.2003.07.016

[69] Nagahama S, Kurihara K. Norepinephrine as a possible transmitter involved in synaptic transmission in frog taste organs and Ca dependence of its release. The Journal of general physiology. 1985;85(3):431–42. 2985736 10.1085/jgp.85.3.431 PMC2215794

[70] Nagai T, Delay RJ, Welton J, Roper SD. Uptake and release of neurotransmitter candidates, [3H]serotonin, [3H]glutamate, and [3H]gamma-aminobutyric acid, in taste buds of the mudpuppy, Necturus maculosus. The Journal of comparative neurology. 1998;392(2):199–208. 9512269

[71] Ogura T. Acetylcholine increases intracellular Ca2+ in taste cells via activation of muscarinic receptors. Journal of neurophysiology. 2002;87(6):2643–9. 12037167 10.1152/jn.2002.87.6.2643

[72] Vandenbeuch A, Tizzano M, Anderson CB, Stone LM, Goldberg D, Kinnamon SC. Evidence for a role of glutamate as an efferent transmitter in taste buds. BMC neuroscience. 2010;11:77 10.1186/1471-2202-11-77 20565975 PMC2898831

[73] Zhang Y, Kolli T, Hivley R, Jaber L, Zhao FI, Yan J, et al Characterization of the expression pattern of adrenergic receptors in rat taste buds. Neuroscience. 2010;169(3):1421–37. 10.1016/j.neuroscience.2010.05.021 20478367 PMC2914163

[74] Zhao FL, Shen T, Kaya N, Lu SG, Cao Y, Herness S. Expression, physiological action, and coexpression patterns of neuropeptide Y in rat taste-bud cells. Proceedings of the National Academy of Sciences of the United States of America. 2005;102(31):11100–5. 16040808 10.1073/pnas.0501988102 PMC1182420

[75] Niki M, Takai S, Kusuhara Y, Ninomiya Y, Yoshida R. Responses to apical and basolateral application of glutamate in mouse fungiform taste cells with action potentials. Cellular and molecular neurobiology. 2011;31(7):1033–40. 10.1007/s10571-011-9702-5 21573975 PMC11498453

[76] Hiji Y, Sato M. [Synergism between 5'-AMP and sodium glutamate on taste receptors of rats]. Nihon seirigaku zasshi Journal of the Physiological Society of Japan. 1967;29(10):602–3. 5627080

[77] Hiji Y, Sato M. [Synergism between 5'-GMP and amino acids on taste receptors of rats]. Nihon seirigaku zasshi Journal of the Physiological Society of Japan. 1967;29(6):274–5. 5625354

[78] Ninomiya Y, Kajiura H, Mochizuki K. Differential taste responses of mouse chorda tympani and glossopharyngeal nerves to sugars and amino acids. Neuroscience letters. 1993;163(2):197–200. 8309632 10.1016/0304-3940(93)90381-t

[79] Ninomiya Y, Kurenuma S, Nomura T, Uebayashi H, Kawamura H. Taste synergism between monosodium glutamate and 5'-ribonucleotide in mice. Comparative biochemistry and physiology A, Comparative physiology. 1992;101(1):97–102. 1347736 10.1016/0300-9629(92)90634-3

[80] Ninomiya Y, Nakashima K, Fukuda A, Nishino H, Sugimura T, Hino A, et al Responses to umami substances in taste bud cells innervated by the chorda tympani and glossopharyngeal nerves. The Journal of nutrition. 2000;130(4S Suppl):950S–3S. 10736359 10.1093/jn/130.4.950S

[81] Sato M, Yamashita S, Ogawa H. Potentiation of gustatory response to monosodium glutamate in rat chorda tympani fibers by addition of 5'-ribonucleotides. The Japanese journal of physiology. 1970;20(4):444–64. 5312071 10.2170/jjphysiol.20.444

[82] Yamamoto T, Matsuo R, Fujimoto Y, Fukunaga I, Miyasaka A, Imoto T. Electrophysiological and behavioral studies on the taste of umami substances in the rat. Physiology & behavior. 1991;49(5):919–25. 1653433 10.1016/0031-9384(91)90204-2

[83] Wifall TC, Faes TM, Taylor-Burds CC, Mitzelfelt JD, Delay ER. An analysis of 5'-inosine and 5'-guanosine monophosphate taste in rats. Chemical senses. 2007;32(2):161–72. 17108183 10.1093/chemse/bjl043

[84] Ninomiya Y, Funakoshi M. Peripheral neural basis for behavioural discrimination between glutamate and the four basic taste substances in mice. Comparative biochemistry and physiology A, Comparative physiology. 1989;92(3):371–6. 2565788 10.1016/0300-9629(89)90578-1

[85] Ninomiya Y, Funakoshi M. Behavioral discrimination between glutamate and the four basic taste substances in mice. Comparative biochemistry and physiology A, Comparative physiology. 1989;92(3):365–70. 2565787 10.1016/0300-9629(89)90577-x

[86] Hellekant G, Danilova V, Ninomiya Y. Primate sense of taste: behavioral and single chorda tympani and glossopharyngeal nerve fiber recordings in the rhesus monkey, Macaca mulatta. Journal of neurophysiology. 1997;77(2):978–93. 9065862 10.1152/jn.1997.77.2.978

[87] Kusakabe Y, Yamaguchi E, Tanemura K, Kameyama K, Chiba N, Arai S, et al Identification of two alpha-subunit species of GTP-binding proteins, Galpha15 and Galphaq, expressed in rat taste buds. Biochimica et biophysica acta. 1998;1403(3):265–72. 9685675 10.1016/s0167-4889(98)00062-7

[88] Sako N, Harada S, Yamamoto T. Gustatory information of umami substances in three major taste nerves. Physiology & behavior. 2000;71(1–2):193–8. 11134701 10.1016/s0031-9384(00)00342-5

[89] Toda Y, Nakagita T, Hayakawa T, Okada S, Narukawa M, Imai H, et al Two distinct determinants of ligand specificity in T1R1/T1R3 (the umami taste receptor). The Journal of biological chemistry. 2013;288(52):36863–77. 10.1074/jbc.M113.494443 24214976 PMC3873546

[90] Zhang F, Klebansky B, Fine RM, Xu H, Pronin A, Liu H, et al Molecular mechanism for the umami taste synergism. Proceedings of the National Academy of Sciences of the United States of America. 2008;105(52):20930–4. 10.1073/pnas.0810174106 19104071 PMC2606899

[91] Kim MR, Kusakabe Y, Miura H, Shindo Y, Ninomiya Y, Hino A. Regional expression patterns of taste receptors and gustducin in the mouse tongue. Biochemical and biophysical research communications. 2003;312(2):500–6. 14637165 10.1016/j.bbrc.2003.10.137

[92] Hoon MA, Adler E, Lindemeier J, Battey JF, Ryba NJ, Zuker CS. Putative mammalian taste receptors: a class of taste-specific GPCRs with distinct topographic selectivity. Cell. 1999;96(4):541–51. 10052456 10.1016/s0092-8674(00)80658-3

[93] Kitagawa M, Kusakabe Y, Miura H, Ninomiya Y, Hino A. Molecular genetic identification of a candidate receptor gene for sweet taste. Biochemical and biophysical research communications. 2001;283(1):236–42. 11322794 10.1006/bbrc.2001.4760

[94] Kusakabe Y, Kim MR, Miura H, Shindo Y, Ninomiya Y, Hino A. Regional expression patterns of T1r family in the mouse tongue. Chemical senses. 2005;30 Suppl 1:i23–4. 15738129 10.1093/chemse/bjh094

[95] Nelson G, Hoon MA, Chandrashekar J, Zhang Y, Ryba NJ, Zuker CS. Mammalian sweet taste receptors. Cell. 2001;106(3):381–90. 11509186 10.1016/s0092-8674(01)00451-2

[96] Delay ER, Beaver AJ, Wagner KA, Stapleton JR, Harbaugh JO, Catron KD, et al Taste preference synergy between glutamate receptor agonists and inosine monophosphate in rats. Chemical senses. 2000;25(5):507–15. 11015322 10.1093/chemse/25.5.507

[97] Sako N, Tokita K, Sugimura T, Yamamoto T. Synergistic responses of the chorda tympani to mixtures of umami and sweet substances in rats. Chemical senses. 2003;28(3):261–6. 12714449 10.1093/chemse/28.3.261

[98] Baillie GL, Horswill JG, Anavi-Goffer S, Reggio PH, Bolognini D, Abood ME, et al CB(1) receptor allosteric modulators display both agonist and signaling pathway specificity. Molecular pharmacology. 2013;83(2):322–38. 10.1124/mol.112.080879 23160940 PMC3558808

[99] Schwartz TW, Holst B. Ago-allosteric modulation and other types of allostery in dimeric 7TM receptors. Journal of receptor and signal transduction research. 2006;26(1–2):107–28. 16595341 10.1080/10799890600567570

[100] Schwartz TW, Holst B. Allosteric enhancers, allosteric agonists and ago-allosteric modulators: where do they bind and how do they act? Trends in pharmacological sciences. 2007;28(8):366–73. 17629958 10.1016/j.tips.2007.06.008

[101] Bystrova MF, Romanov RA, Rogachevskaja OA, Churbanov GD, Kolesnikov SS. Functional expression of the extracellular-Ca2+-sensing receptor in mouse taste cells. Journal of cell science. 2010;123(Pt 6):972–82. 10.1242/jcs.061879 20179105

[102] San Gabriel A, Uneyama H, Maekawa T, Torii K. The calcium-sensing receptor in taste tissue. Biochemical and biophysical research communications. 2009;378(3):414–8. 10.1016/j.bbrc.2008.11.060 19056349

